# MicroRNA-34a Encapsulated in Hyaluronic Acid Nanoparticles Induces Epigenetic Changes with Altered Mitochondrial Bioenergetics and Apoptosis in Non-Small-Cell Lung Cancer Cells

**DOI:** 10.1038/s41598-017-02816-8

**Published:** 2017-06-16

**Authors:** Malav Trivedi, Amit Singh, Meghna Talekar, Grishma Pawar, Parin Shah, Mansoor Amiji

**Affiliations:** 1 Department of Pharmaceutical Sciences, School of Pharmacy, Northeastern University, Boston, Massachusetts 02115 USA; 2 Department of Pharmaceutical Sciences, School of Pharmacy, Nova Southeastern University, Davie, Florida 33317 USA; 3 Faculty of Pharmacy, King Abdulaziz University, Jeddah, Saudi Arabia

## Abstract

Therapies targeting epigenetic changes for cancer treatment are in Phase I/II trials; however, all of these target only nuclear DNA. Emerging evidence suggests presence of methylation marks on mitochondrial DNA (mtDNA); but their contribution in cancer is unidentified. Expression of genes encoded on mtDNA are altered in cancer cells, along with increased glycolytic flux. Such glycolytic flux and elevated reactive oxygen species is supported by increased antioxidant; glutathione. MicroRNA-34a can translocate to mitochondria, mediate downstream apoptotic effects of tumor suppressor P53, and inhibit the antioxidant response element Nrf-2, resulting in depleted glutathione levels. Based on such strong rationale, we encapsulated microRNA-34a in our well-established Hyaluronic-Acid nanoparticles and delivered to cisplatin-sensitive and cisplatin-resistant A549-lung adenocarcinoma cells. Successful delivery and uptake in cells resulted in altered ATP levels, decreased glycolytic flux, Nrf-2 and glutathione levels, ultimately resulting in caspase-3 activation and apoptosis. Most important were the concurrent underlying molecular changes in epigenetic status of D-loop on the mtDNA and transcription of mtDNA-encoded genes. Although preliminary, we provide a novel therapeutic approach in form of altered mitochondrial bioenergetics and redox status of cancer cells with underlying changes in epigenetic status of mtDNA that can subsequently results in induction of cancer cell apoptosis.

## Introduction

Bioenergetic complexities at the cellular and subcellular level in lung cancer cells are suitably complemented with molecular fingerprints permissive for drug resistance, cell survival and inhibition of various apoptotic pathways^1, 2^. One such cancer specific feature is a defective mitochondrial function complemented by a predominant metabolic switch towards glycolytic-ATP production^3^. Similarly, an elevated level of antioxidant response element like nuclear erythroid factor (NRF2) and elevated antioxidants such as glutathione (GSH) is another such metabolic switch in proliferating cancer cells^4–7^. Such metabolic changes are supported by molecular adaptations. Although such molecular fingerprints are generally attributed to genetic mutations in cancer genome [both nuclear DNA (nDNA) and mitochondrial DNA (mtDNA)]^8–12^; substantial evidence indicates that epigenomic contribution to cancer development and progression is equally or probably even more prominent in supporting cancer cell survival and aggressive behavior^13–16^.

Although epigenetic-based therapeutic modalities are already in pre-clinical and clinical trials, such therapies are targeted towards nDNA^13, 16, 17^ and not on mtDNA. Thirteen core essential oxidative phosphorylation (OXPHOS) genes for mitochondrial protein synthesis are encoded on the mtDNA^18^; however in cancer cells, most of the mitochondrial function is decreased and mtDNA transcription is deregulated^3, 19^. Although depletion of mtDNA^20^ and characteristic mutations/deletions in control and coding regions of mtDNA are commonly identified with lung cancer progression and aggressiveness^21, 22^; the role of epigenetic changes on mtDNA in lung cancer is not examined. Emerging evidence indicates that DNA methyltransferase (DNMT) responsible for methylation and Ten-Eleven-Translocase (TET) enzymes that are responsible for demethylation of CpG site on nDNA, can also be translocated to mitochondria^23–25^. Both, the methylation marker: 5-methylcytosine (5mC) and demethylation marker: 5-hydroxymethylcytosine (5hmC), are observed at the CpG site on mtDNA same as nDNA^24–27^.

Tumor suppressor p53 can also support mitochondrial functioning^28–30^ and absence of p53 (associated with cancer cells) can directly upregulate the levels of DNMT1 and mtDNMT1 as well as alter epigenetic status at specific gene sites on mtDNA^23^. MicroRNA-34a (miR34a) is responsible for several downstream effects of p53 but is downregulated in some human cancers, including lung cancer^31–33^. miR34a overexpression can limit cancer cell growth and tumor progression in NSCLC models^34, 35^. miR34a also antagonizes many different oncogenic processes by regulating genes that function in various cellular pathways (i.e., Wnt1, Notch1, Wnt3, MTA2, CD44, c-MYC among others)^34, 36^. Recent studies suggest that miR34a is also known to be expressed in the mitochondria^37, 38^. However, it is not known if miR34a can induce any effects on levels of DNMT1, mtDNMT1 and alter epigenetic status on mtDNA.

Among the various epigenetic therapies for cancer treatment, the use of nucleic acid constructs as therapeutics has enormous potential, but their delivery using viral-based delivery systems has several challenges, including the risk of immunogenicity^39^. Nanoparticle (NP) based drug delivery for nucleic acid therapies (NATs) can improve drug loading, enhance drug delivery, reduce toxicity and decrease immunogenicity. Previously we demonstrated the successful use of novel drug delivery system incorporating self-assembling hyaluronic acid-poly(ethylene imine) (HA-PEI) and HA-poly(ethylene glycol) (HA-PEG) blend nanoparticles for the delivery of small interfering RNA in A549 cells^40^. Here, we have extended such nanoparticle application for the delivery of miR34a in A549 human lung adenocarcinoma epithelial cell line for redox-epigenetic modifications. We investigated if miR34a can alter nuclear and mitochondrial epigenetic enzymes and induce epigenetic and transcriptional changes on nuclear as well as mtDNA. Concurrently, we also characterized if such underlying epigenetic/transcriptional changes can contribute to altered mitochondrial bioenergetics and redox status of cancer cells subsequently leading to cancer cells’ apoptosis.

## Results

### Formulation characterization of miR34a encapsulated HA-PEI/HA-PEG nanoparticles (miR34a HA-NPs)

**Figure 1:**
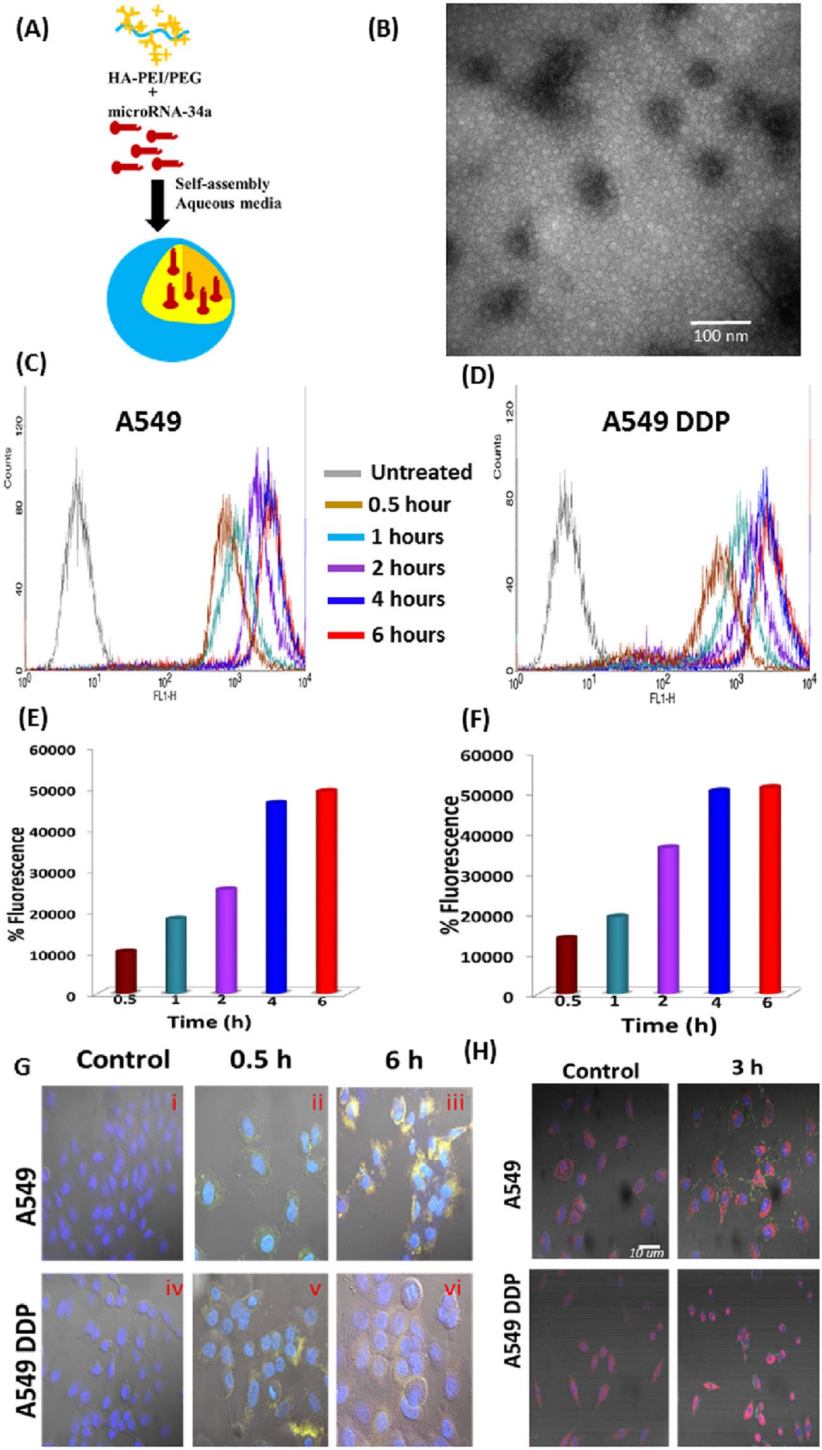
Characteristics of miR34a hyaluronic acid (HA)-based nanoparticles. (**A**) Schematic representation of HA-PEI/HA-PEG nano-assemblies encapsulated with miR34a duplexes. **(B)** The self-assembling miR34a HA-NPs nanoparticles showed a spherical morphology in TEM. **(C–F)** Flow cytometry analysis indicated that the miR34a HA-NPs were taken up by the A549 cells and cisplatin resistant A549 cells (A549 DDP) cells in a time dependent manner. The HA-PEI was conjugated with rhodamine dye and the miR34a was conjugated with FITC. **(G**,**H)** Confocal images to support the flow cytometry results indicating the uptake of the FITC-miR34a encapsulated in HAPEI nanovectors; mitochondria stained by MitoTrackr™.

We have previously reported the synthesis and targeted delivery of HA-NPs in SKOV-3 ovarian adenocarcinoma cells and SK-LU-1 lung adenocarcinoma cells^41, 42^. In this study, we encapsulated miR34a duplexes in these self-assembling HA-NPs (Fig. 1A). These NPs had a spherical morphology (Fig. 1B) with an average size of 260–360 nm and surface charge of −35 mV. In addition, as previously described^42^, we were able to achieve 94–96% encapsulation efficiency for miR34a during different batches of formulation (Fig. S10).

### Qualitative and quantitative assessment of miR34a HA NP uptake in cisplatin sensitive A549 and cisplatin resistant A549 DDP cells

Cellular uptake studies were conducted using flow cytometry with rhodamine-labeled HA-PEI and FITC labeled miR34a for the preparation of the HA-NPs. These labeled NPs (50 nM) were incubated with A549 and A549 DDP cells for 30 mins to 6 hours (Fig. 1C–F and Supplementary Figs S1 and S2), which showed a consistent uptake of the rhodamine-labeled HA-NPs as well as FITC-labeled miR34a in both A549 and A549 DDP cells. A 4–5 fold increase in the levels of miR34a were observed even after 4 hours of incubation in both A549 and A549 DDP cells (Fig. 1C–F).

To investigate the cellular and organelle-specific localization of miR34a-encapsulated nanoparticles we performed qualitative confocal microscopy with FITC-labeled miR34a encapsulated in HA-NPs (Fig. 1G and Supplementary Figs S3 and S4). As indicated, cellular uptake of FITC-miR34a was observed within 30 mins of incubation in both; A549 (Fig. 1Gi–Giii) and A549 DDP cell lines (Fig. 1Giv–Gvi). The miR34a was present at least until six hrs of incubation. We also used MitoTracker™ Red mitochondrial staining along with miR34a HA-NPs and observed that miR34a was also present in the mitochondria as well as in the cytoplasm (Fig. 1H and Supplementary Figs S5 and S6). Hence, our nanoparticles successfully transfected both A549 and A549 DDP and provide sustained elevated levels of intracellular miR34a. Although we did not target our nanoparticles to the mitochondria, the miR34a was present in low levels in mitochondria (Fig. 1H and Supplementary Figs S5 and S6). It is important to note that we have previously reported no differences in cellular uptake and toxicity with blank with nanoparticles only encapsulating Rhodamine or FITC dyes^40, 42–44^.

### Induction of apoptosis in A549 and A549 DDP cells using miR34a HA-NPs

**Figure 2:**
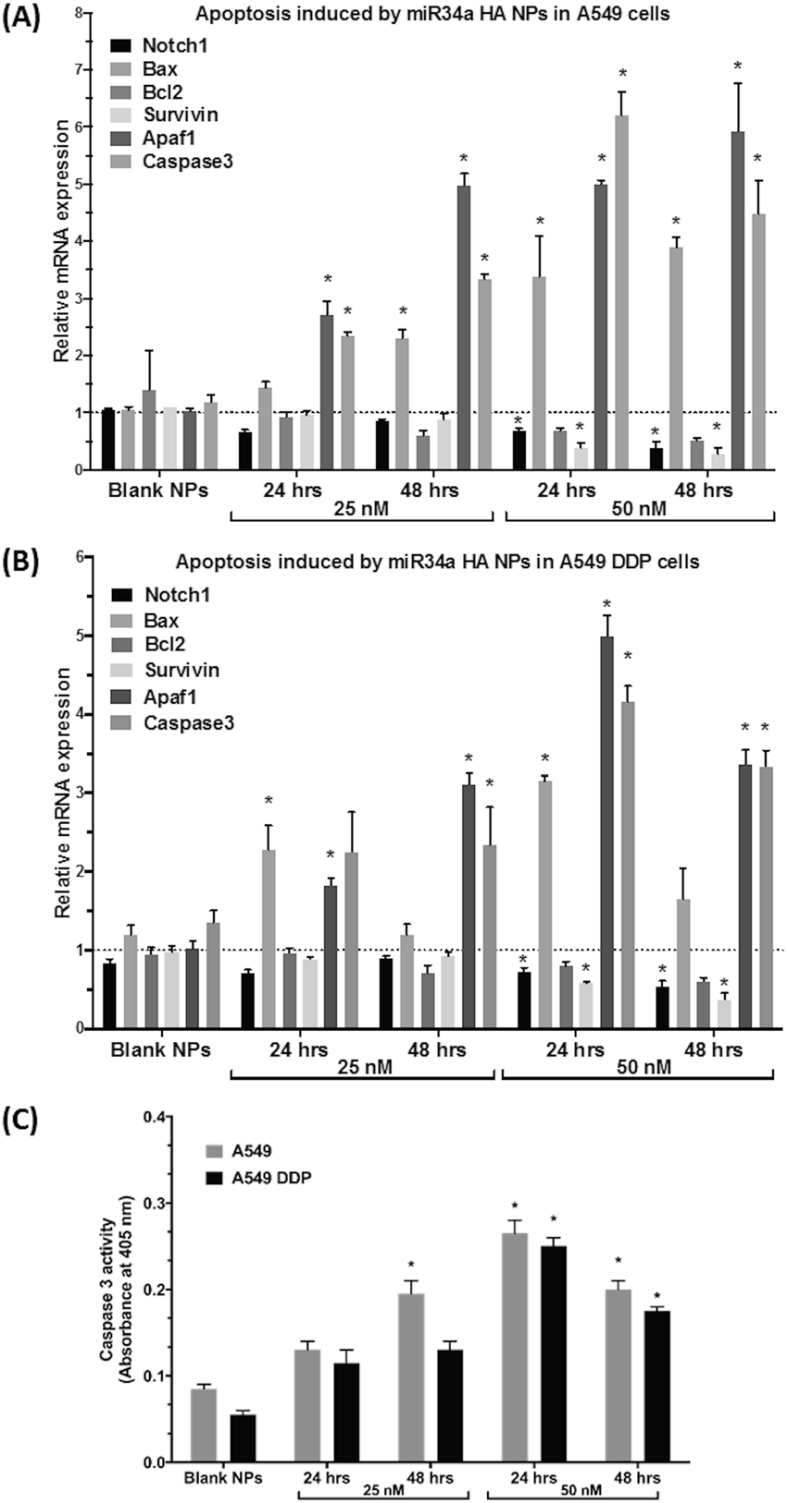
MicroRNA-34a induced cellular apoptosis. Dose-dependent and time-dependent analyses of genes responsible for induction of apoptotic signaling and simultaneous inhibition of anti-apoptotic genes, post transfection with HA-PEI/HA-PEG-nanoparticles encapsulated miR34a on **(A)** wild-type A549 and **(B)** cisplatin resistant A549DDP cells. Normalized to beta-actin and untreated control samples using relative quantification by delta(delta(Ct) method. **(C)** Caspase 3 activity levels measured using ELISA assay in both A549 and A549 DDP cells. Blank NPs–Blank HA-PEI/HA-PEG nanoparticles, miR34a HA-NPs–microRNA34a encapsulated in HA-PEI/HA-PEG nanoparticles. *Indicates comparison against control = 1. One-way ANOVA followed by Post-hoc t-test with multiple comparisons, *p < 0.05, N = 6.

Next, we evaluated the functional consequences of a successful treatment of miR34a HA-NPs. A549 and A549 DDP cells were transfected with miR34a HA-NPs (25 and 50 nM) for 24 and 48 hrs and apoptotic gene expression was measured. Downstream pro-apoptotic transcription factors Bax, Apaf-1, PUMA, caspase3 as well as anti-apoptotic factors like Bcl-2, survivin were analyzed with qRT-PCR. Relative changes were compared to untreated control levels for A549 (Fig. 2A) and A549DDP cells (Fig. 2B). The results indicated that miR34a HA-NPs induced a significant dose and time-dependent increase in all the pro-apoptotic factors in both A549 (Fig. 2A) and A549 DDP cells (Fig. 2B). In contrast, significant decline in the anti-apoptotic factors; namely, Bcl2 and survivin was observed in both A549 (Fig. 2A) and A549 DDP cells (Fig. 2B). Scrambled miRNA, HA-PEI and naked miR34a had no/moderate but not significant effect (Fig. 2A,B). Hence, it was observed that miR34a HA-NPs could induce apoptosis in both A549 and A549 DDP NSCLC cell lines. Since treatment with miR34a at 50 nM for duration of 24 hrs provided an effective induction of apoptosis in both A549 and A549 DDP cells further studies were conducted under these treatment conditions.

We further validated this dose by measuring the apoptotic activation and characterized if changes in the expression of pro- and anti-apoptotic genes, actually translated into changes in the apoptosis levels. Hence, we measured the caspase-3 activity in both A549 and A549 DDP cells after treatment with miR34a HA-NPs. As indicated in Fig. 2C, treatment with miR34a HA-NPs (50 nM for 24 and 48 hrs) induced caspase-3 activity in both A549 and A549 DDP cells. However, such changes were not observed at 25 nM. Furthermore, no caspase activation was observed with blank HA-NPs (Fig. 2C). Hence, miR34a HA-NPs induced apoptosis by elevating caspase-3 activity, as well as other pro-apoptotic genes and decreasing the levels of anti-apoptotic genes.

### Alteration of mitochondrial epigenetic enzymes following treatment with miR34a HA-NPs

**Figure 3:**
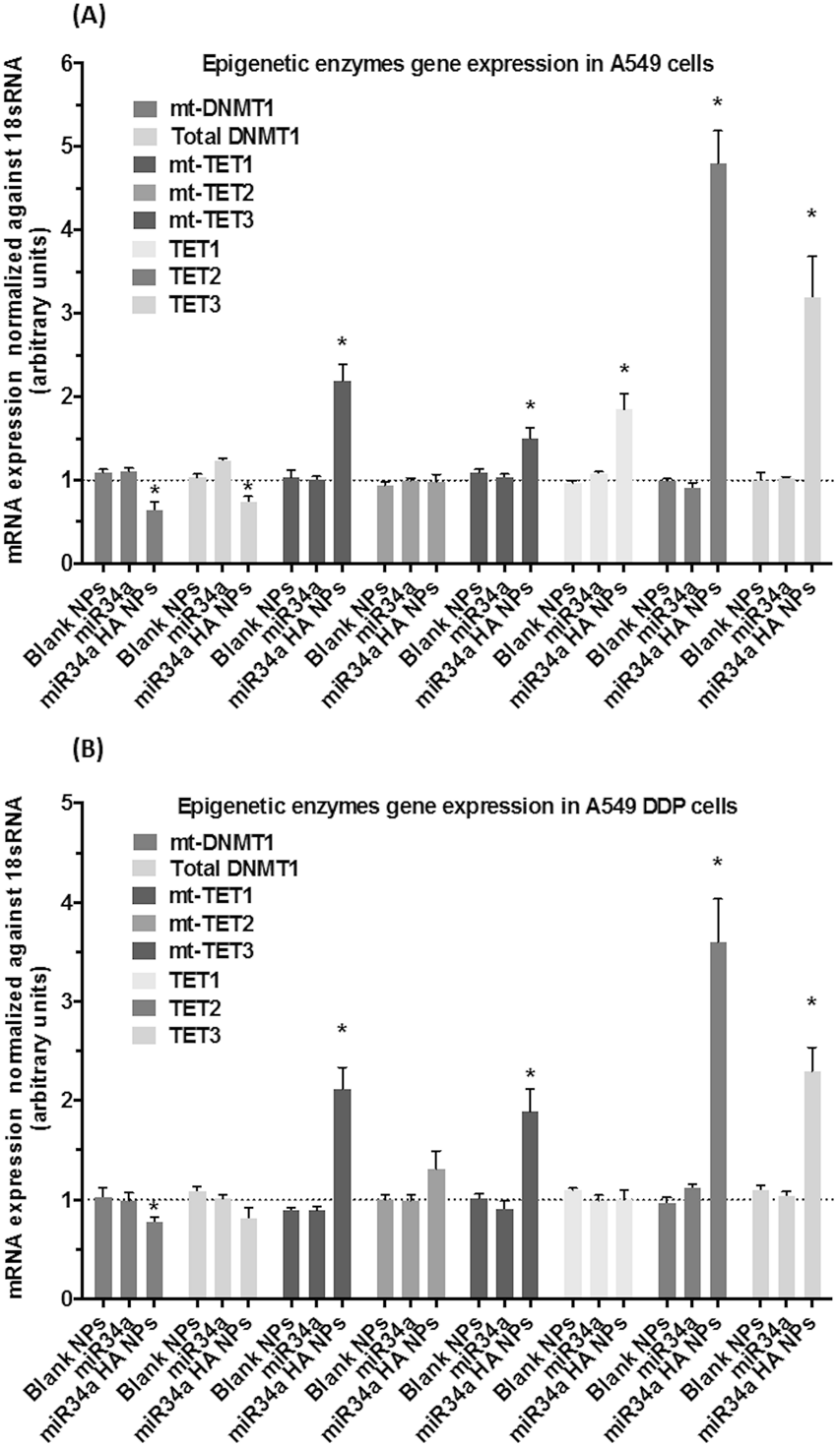
Effect of miR34a transfection with HA-based nanoparticles on mRNA content of epigenetic enzymes in cellular and mitochondrial (mt) compartment. A quantitative real time polymerase chain reaction (qRT-PCR) assay was for measurement of epigenetic enzyme levels in (**A**) wild-type A549 and (**B**) cisplatin resistant A549DDP cells post-transfection with HA-PEI/PEG-nanovectors encapsulated miR34a. The levels were normalized to beta-actin for nuclear genes and 18 s ribosomal RNA for mitochondrial genes, as well as untreated A549 or A549 DDP control cells using relative quantification by delta(delta(Ct) method. Mitochondrial DNA methyltransferase (mt-DNMT1), Total-DNA methyltransferase (Total-DNMT1), mitochondrial Ten-Eleven-Translocase 1–3 (mt-TET1–3), total TET1–3 (TET1–3). Blank NPs–Blank HA-PEI/HA-PEG Nanoparticles, miR34a - Naked miR34a without encapsulation, miR34a HA-NPs–microRNA34a encapsulated in HA-PEI/HA-PEG nanoparticles. Results are expressed in units (mean +/− standard error of mean [SEM]); *indicates comparison against control = 1. One-way ANOVA followed by Post-hoc t-test with multiple comparisons, *p < 0.05; N = 6.

Both preclinical and clinical studies report an elevated DNMT1 levels in several tumors including lung cancer^45–48^. Furthermore, the loss of p53 can up-regulate the levels of DNMT1 and mtDNMT1^23^. Since miR34a is downstream of p53, we evaluated if miR34a HA-NPs can alter DNMT1 and mtDNMT1 levels in the nucleus and mitochondria respectively, of A549 and A549 DDP cell lines. As depicted in Fig. 3, treatment with miR34a HA-NPs (50 nM, 24 hrs) resulted in decreased mRNA levels of both DNMT1 and mtDNMT1 in both A549 (Fig. 3A) as well as A549 DDP cell lines (Fig. 3B), as compared to the untreated controls. The DNMT1 levels were decreased by 15–20% in both A549 and A549 DDP cells, whereas mtDNMT1 levels decreased by 30–40% only in A549 cells. Hence, these results suggest a contributive role of miR34a in p53-mediated regulation on DNMT1 and mtDNMT1.

The TET1–3 enzymes, which are responsible for the oxidation of 5-mC to 5-hmC are identified as marks for active DNA demethylation and also reported to be present in the mitochondria^24–26^. Hence, we investigated if miR34a HA-NPs (50 nM, 24 hrs) can induce any effects on total TET1–3 and mt-TET1–3 enzyme levels. Figure 3 shows elevated mRNA levels of mt-TET1 and mt-TET3 in A549 (Fig. 3A) and A549 DDP cells (Fig. 3B) after treatment with miR34a HA-NPs. The mt-TET1 mRNA levels were almost doubled in both A549 (Fig. 3A) and A549 DDP (Fig. 3B); under the influence of miR34a HA-NPs (50 nM, 24 hrs) with 30 and 50% increase in mt-TET3 levels in A549 (Fig. 3A) and A549 DDP cells (Fig. 3B), respectively. However, no significant changes were observed with mt-TET2 levels. In contrast, total TET2 levels in the nucleus were elevated along with significant elevation in TET3 in both A549 (Fig. 3A) and A549 DDP (Fig. 3B). This is consistent with the previous studies suggesting the contribution of TET1–3 towards cancer progression and metastasis^49, 50^.

### Modification in epigenetic status

Next, we investigated if reductions observed in DNMT1 and mtDNMT1 as well as elevation in TET1–3 and mt-TET1–3 translated in any changes in methylation status of nDNA and mtDNA.

#### Alteration in the nDNA methylation status

**Figure 4:**
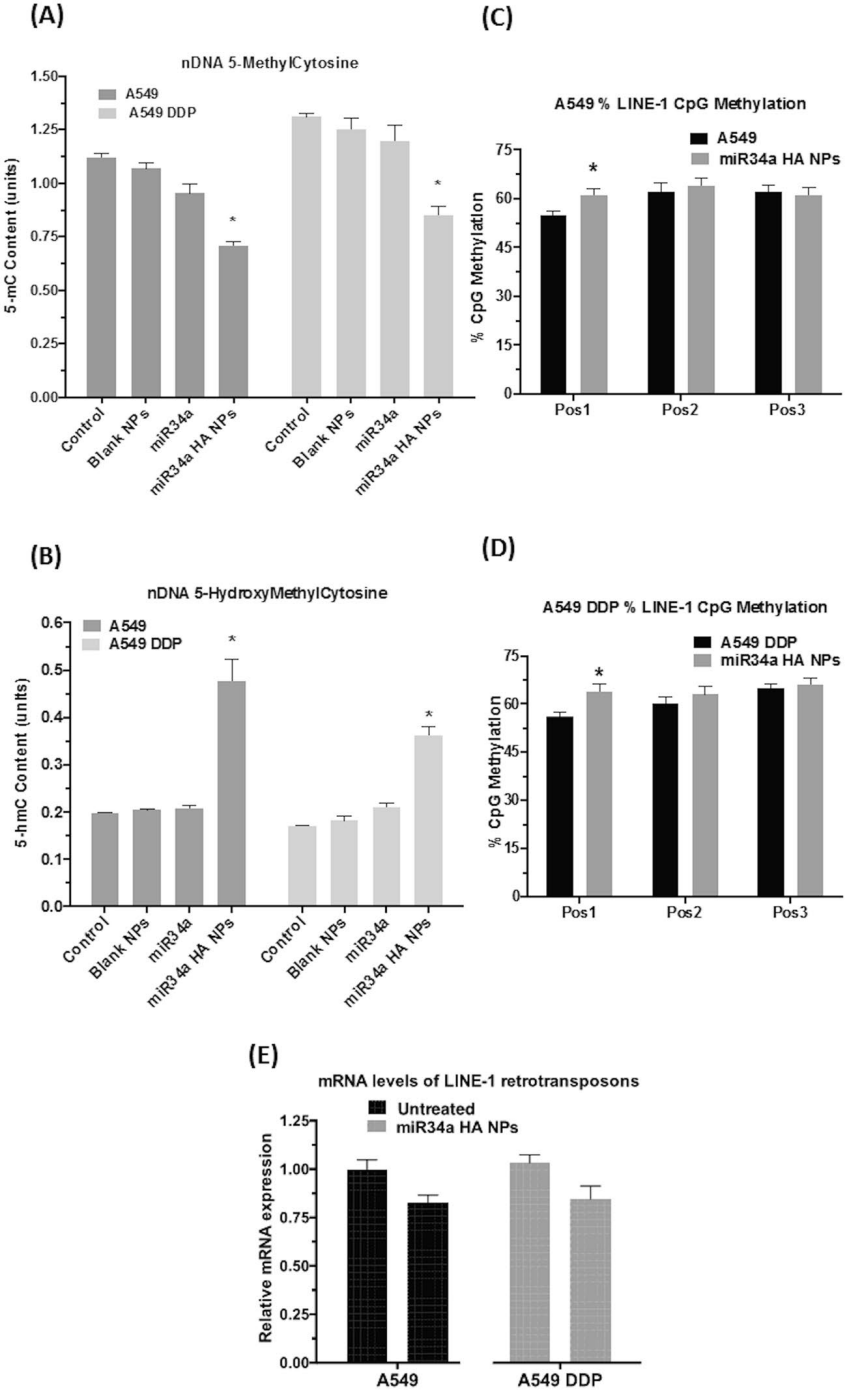
Effect of miR34a transfection with HA-based nanoparticles on nuclear DNA (nDNA) methylation levels. Nucleus was isolated from A549 and A549 DDP cells post-transfection with miR34a HA-NPs. DNA was isolated from this nucleus and global **(A)** 5-methylcytosine (5-mC); **(B)** 5-hydroxymethylcytosine (5-hmC) content were measured with equal amounts of nDNA using 5-mC and 5-hmC enzyme linked immunosorbent assay (ELISA) assays. Blank NPs–Blank HA-PEI/HA-PEG nanoparticles, miR34a - Naked miR34a without encapsulation, miR34a HA-NPs–microRNA34a encapsulated in HA-PEI/HA-PEG nanoparticles. **(C**,**D)** miR34 a HA-NPs induced changes in site-specific methylation content for individual CpG sites on the promoter regions of LINE-1Hs family as determined by bisulfite conversion followed by pyrosequencing. Pos1–3 indicates individual CpG sites. Results are expressed in units (mean +/− standard error of the mean [SEM]); N = 6; *p < 0.05 compared to the corresponding control. (**E**) mRNA levels of LINE-1 retrotransposons as measured by a quantitative real time polymerase chain reaction (qRT-PCR) assay on the cells post-transfection with HA-PEI/PEG-nanovectors encapsulated miR34a. Normalized to Beta-Actin as well as untreated A549 or A549 DDP control cells using relative quantification by delta(delta(Ct) method. Results are expressed in units (mean +/− standard error of the mean [SEM]; n = 6).

Global nDNA methylation levels were measured using anti–5-methylcytosine and anti-5-hydroxymethylcytosine monoclonal antibodies, quantified by an enzyme-linked immunosorbent assay–like reaction (ELISA) as described in methods. In both A549 and A549 DDP cells, global 5-mC levels decreased by about ~25% post-treatment with miR34a HA-NPs (50 nM, 24 hrs; p < 0.05). However, in contrast, the global 5-hmC levels were significantly elevated (p < 0.05) as compared to untreated cells (Fig. 4A,B). No significant changes were observed with naked miR34a or blank nanoparticles.

Long interspersed nuclear elements (LINE1; L1 elements) are retrotransposons spread across the entire human genome^51^. Changes in L1 methylation and L1 genomic activity have also been associated with tumor progression and development^52–55^. Using bisulfite conversion followed by a pyrosequencing, we have previously reported alterations in the levels of L1 methylation under the effect of various drugs^56^. Following a similar procedure, we measured the methylation levels at 3 different CpG sites in the open reading frame 1 (ORF-1) of L1 retrotransposon. At CpG Position 1, treatment with miR34a HA-NPs (50 nM, 24 hrs) induced an increase in levels of CpG methylation (62 +/− 0.78, 61 +/− 0.81)% on L1 retrotransposons as compared to untreated A549 and A549 DDP cells (55 +/− 0.63, 56 +/− 0.73)% respectively (p < 0.05; N = 6) (Fig. 4C,D). No significant changes were observed at the other 2 CpG sites. It should also be noted that bisulfite conversion and pyrosequencing does not differentiate between 5mC and 5hmC and hence the above values can be indicative of both of these methylation state.

To determine whether the above changes in LINE-1 CpG methylation were associated with changes in transcription of LINE1, we designed primers specific for the L1-Hs family and quantified the respective mRNA levels using qRT-PCR as previously described. Treatment with miR34a HA-NPs (50 nM, 24 hrs) decreased L1 mRNA levels; however, this decline was not significant (Fig. 4E). Thus, although the levels of DNMT1 decreased as well as global DNA 5mC methylation levels were subsequently decreased, the methylation levels at the position 1 CpG site were significantly increased, but the L1 mRNA levels did not alter. This is not surprising especially since p53 is the guardian of the genome and p53 is known to employ L1 retrotransposons as a DNA damaging agent for signaling apoptosis^52, 57–59^. However, in this case miR34a did not induce elevated levels of L1 retrotransposon, indicating it might be an L1-independent induction of apoptosis.

#### Alteration in the mtDNA methylation status

**Figure 5:**
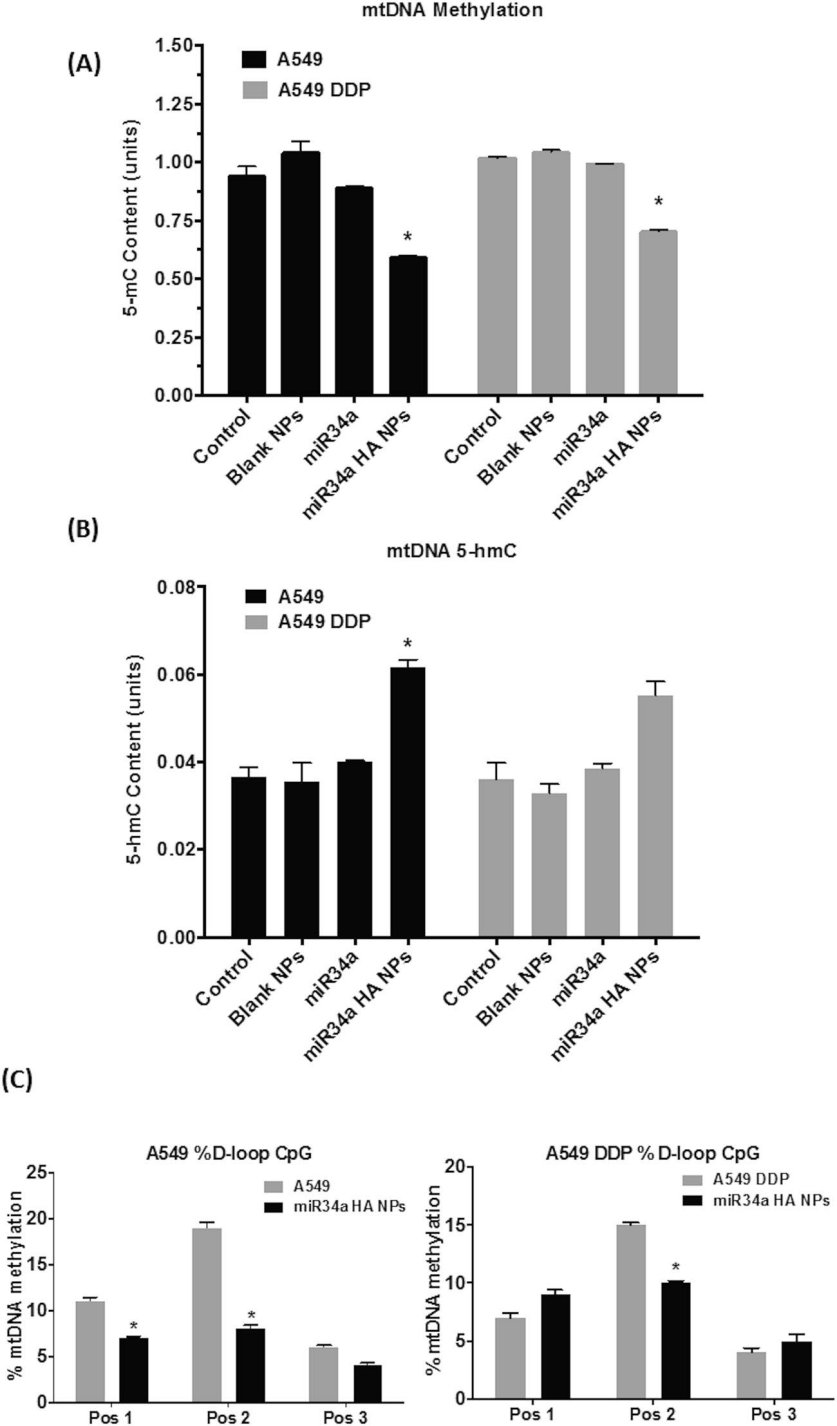
Effect of miR34a transfection with HA-based nanoparticles on mitochondrial DNA (mtDNA) Methylation levels. Mitochondria was isolated from A549 and A549 DDP cells post-transfection with miR34a HA-NPs (50 nM; 24 hours) DNA was isolated from this mitochondria and global **(A)** 5-methylcytosine (5-mC) **(B)** 5-hydroxymethylcytosine (5-hmC) content were measured with equal amounts of mtDNA using 5-mC and 5-hmC enzyme linked immunosorbent assay (ELISA) assays. Blank NPs–Blank HA-PEI/HA-PEG nanoparticles, miR34a - Naked miR34a without encapsulation, miR34a HA-NPs–microRNA34a encapsulated in HA-PEI/HA-PEG nanoparticles. **(C)** miR34 a HA-NPs induced changes in site-specific methylation content for individual CpG sites on the promoter regions of D-loop on mt-DNA as determined by bisulfite conversion followed by pyrosequencing. Pos1–3 indicates individual CpG sites. Results are expressed in units (mean +/− standard error of the mean [SEM]); n = 6; *p < 0.05 compared to the corresponding control.

Only few studies have been published about mtDNA methylation levels, and the exact role of mtDNA epigenetic status in cancer is still not clear. Global mtDNA methylation levels were measured using the same ELISA kit as nDNA methylation levels. In both A549 and A549 DDP cells, global mtDNA 5-mC levels decreased by about ~30% post- treatment with miR34a HA-NPs (50 nM, 24 hrs). However, in contrast, the global mtDNA 5-hmC levels were significantly elevated by 20% as compared to untreated cells (Fig. 5A,B). No significant changes were observed with naked miR34a or blank nanoparticles.

The D-loop on the mtDNA contains three promoter sequences for initiation of mtDNA transcription. Several previous studies have reported the identification specific CpG sites in this promoter region of D-loop^27, 60–62^. Based on these studies, we evaluated the site-specific CpG methylation on D-loop of mtDNA after treatment with miR34a HA-NPs (50 nM, 24 hrs). The mean mtDNA methylation on the D-loop after treatment with miR34a nanoparticles in A549 cells was (7.0 +/− 0.4%; 8.0 +/− 0.4%; N = 6) as compared to (11.0 +/− 0.5%; 19.0 +/− 0.6%; N = 6) at CpG-1 and CpG-2; respectively, in untreated A549. In contrast, post-treatment with miR34a HA-NPs (50 nM, 24 hrs) of A549 DDP cells did not show any changes on CpG-1 site but had altered CpG-2 site (10.0 +/− 0.3%) as compared to untreated A549 DDP controls (15.0 +/− 0.4%). The functional implication of this small change in D-loop methylation and its significance in the mitochondrial transcription and/or resulting metabolic functioning is not directly investigated; however, the treatment with miR34a HA-NPs alters this D-loop methylation is highly intriguing for future therapeutic modality.

### Gene-specific changes in mitochondrial transcription post-treatment with miR34a HA-NPs

#### Gene-Specific Changes in mtDNA encoded genes

**Figure 6:**
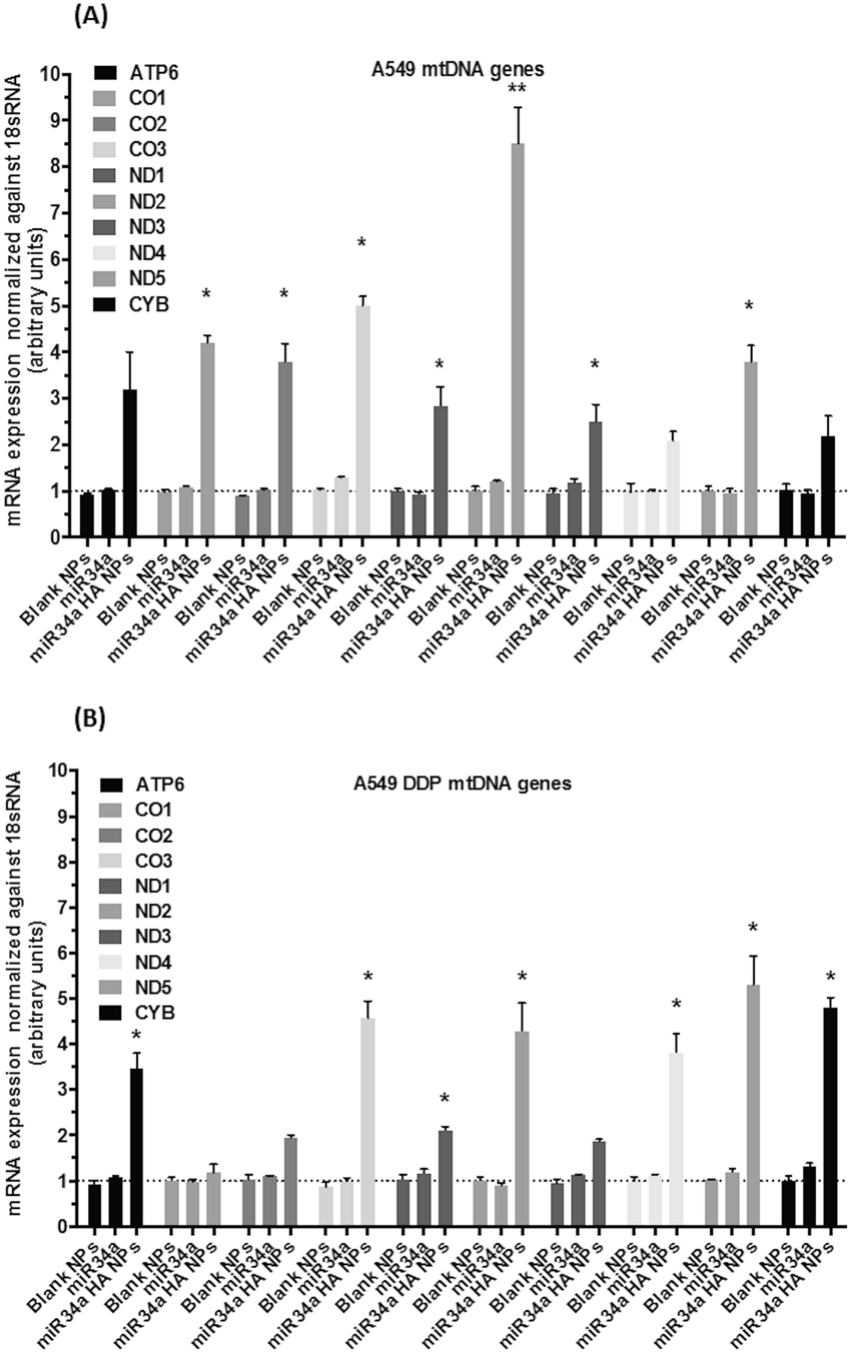
Effect of miR34a transfection with HA-based nanoparticles on mRNA content of mitochondrial DNA (mtDNA) encoded genes. A quantitative real time polymerase chain reaction (qRT-PCR) assay was used with **(A)** wild-type A549 and **(B)** cisplatin resistant A549DDP cells post-transfection with HA-PEI/HA-PEG-nanovectors encapsulated miR34a. Normalized to 18 s ribosomal RNA and untreated control samples using relative quantification by delta(delta(Ct) method. Blank NPs–Blank HA-PEI/HA-PEG nanoparticles, miR34a - Naked miR34a without encapsulation, miR34a HA-NPs–microRNA34a encapsulated in HA-PEI/HA-PEG nanoparticles. Results are expressed in units (mean +/− standard error of mean [SEM]); *indicates comparison against control = 1. One-way ANOVA followed by Post-hoc t-test with multiple comparisons, *p < 0.05, **p < 0.01; n = 6.

Next, we investigated whether miR34a-induced effects on the mtDNA methylation at the D-loop had any consequence on the mRNA transcript levels of mtDNA-encoded genes. We treated A549 and A549 DDP cells with miR34a HA-NPs (50 nM for 24 hrs) and measured the mRNA levels for 10 out of 13 protein coding gene transcripts. Primers specific for mitochondrial-encoded genes were designed and mRNA levels were quantified using qRT-PCR. On the heavy (H) strand, ATPase subunit-6 (ATP6), cytochrome c oxidase subunit 1–3 (CO1–3) and NADH dehydrogenase subunits (ND1-ND5) were also investigated. The miR34a HA-NPs (50 nM, 24 hrs) treatment induced significantly elevated mRNA transcripts of ND1, ND2, ND3, ND5, CO1, CO2, and CO3 as compared to untreated A549 cells (Fig. 6A). In contrast, in the A549 DDP cells, miR34a HA-NPs (50 nM, 24 hrs) treatment induced elevated levels of ATP6, ND1, ND2, ND4, ND5, CO3 and CYB as compared to untreated A549 DDP cells (Fig. 6B). The ND1 is the first H strand protein-coding region following the ribosomal RNA genes and it was significantly increased in response to miR34a HA-NPs treatment in only A549 cells but not A549 DDP cells. Moreover, NADH dehydrogenase subunit 6 (ND6), the only protein-coding gene on the light (L) strand, did not change in response to miR34a-HA-NPs treatment in either A549 or A549 DDP cells (data not shown). Further, no changes were observed with naked miR34a or HA-NPs in either cell lines.

#### Gene-specific changes in nDNA encoded mitochondrial genes

**Figure 7:**
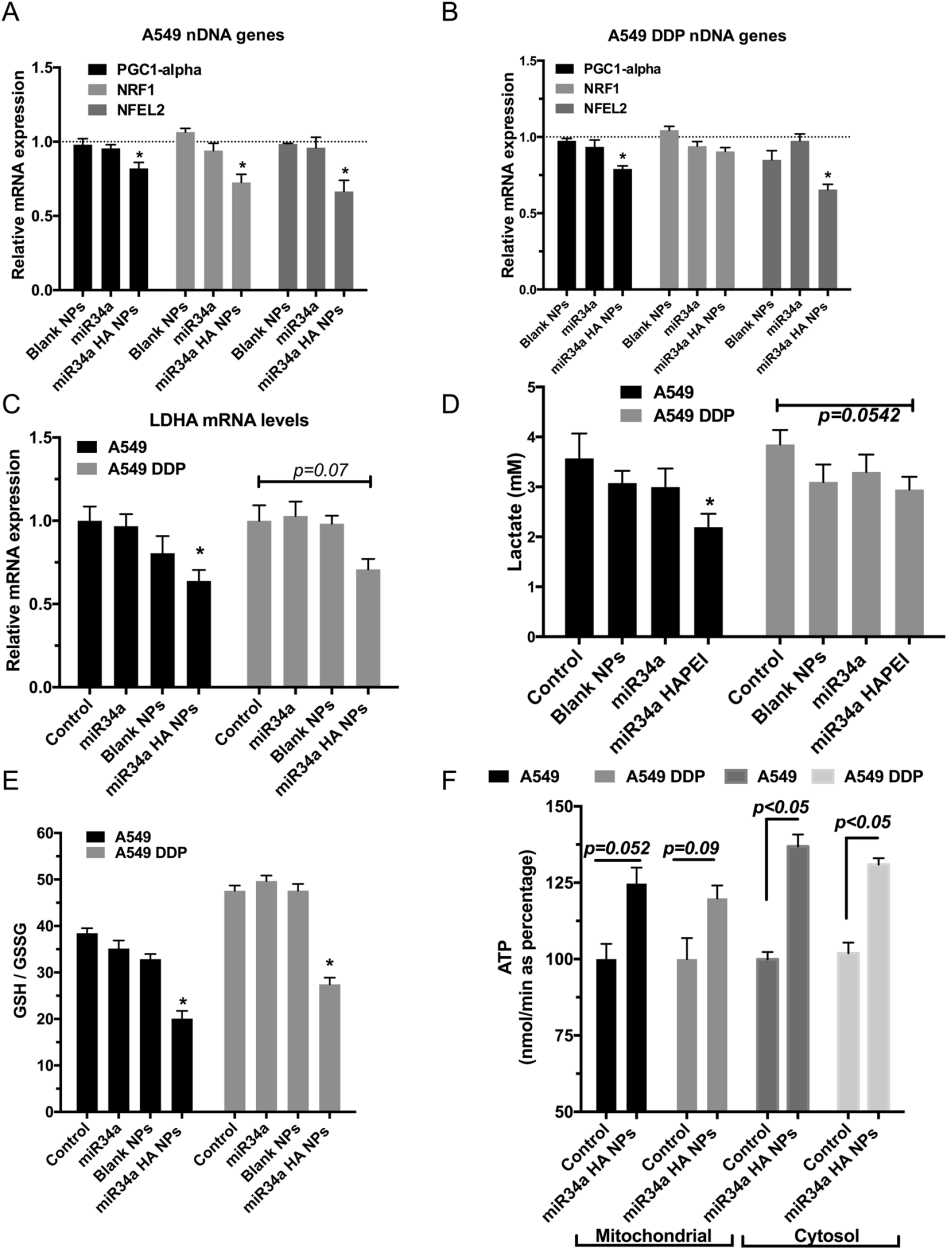
Effect of miR34a transfection with HA-based nanoparticles (50 nM; 24 hours) on mitochondrial bioenergetics and mitochondrial genes encoded on nuclear DNA. A quantitative real time polymerase chain reaction (qRT-PCR) assay was used to measure the levels of PGC1alpha, NRF1 and NRF2 with the (**A**) wild-type A549 cells and (**B**) cisplatin resistant A549DDP cells post-transfection with HA-PEI/HA-PEG-nanovectors encapsulated miR34a, normalized to beta-actin RNA and untreated control samples using relative quantification by delta(delta(Ct) method. (**C**) Lactate dehydrogenase mRNA levels were measured using a qRT-PCR. (**D**) Lactate activity was measured using an ELISA assay. (**E**) Ratios of reduced to oxidized Glutathione levels measured using HPLC. (**F**) Mitochondrial and Cytoplasmic ATP levels measured using ELISA assay. Blank NPs–Blank HA-PEI/HA-PEG nanoparticles, miR34a - Naked miR34a without encapsulation, miR34a HA-NPs–microRNA34a encapsulated in HA-PEI/HA-PEG nanoparticles. Results are expressed in units (mean +/− standard error of mean [SEM]); *indicates comparison against control = 1. One-way ANOVA followed by Post-hoc t-test with multiple comparisons, *p < 0.05, **p < 0.01; n = 6.

Peroxisome proliferator-activated receptor gamma coactivator 1-alpha (PGC-1α), Nuclear erythroid factor-2 (NRF2), and Nuclear respirator Factor 1 (Nrf-1) are few major regulators for mitochondrial functioning that are tightly regulated by p53^63–65^. We investigated the effect of miR34a HA-NPs on these mRNA transcripts and observed a significant decline in the PGC1alpha as well as decreased NRF1 and NRF2 levels, when compared to untreated A549 cells (Fig. 7A). Similarly, in A549 DDP cells, miR34a HA-NPs treatment resulted in decreased PGC1alpha as well as NRF2 levels, however no changes were observed in the NRF1 levels (Fig. 7B).

### Mitochondrial Complex I and Complex IV levels and enzyme activity

In parallel to gene expression studies, we also conducted analysis of NADH dehydrogenase from Complex I and Cytochrome C levels from Complex IV using ELISA assay. The Complex IV levels as well as the enzyme activity were elevated after treatment with miR34a HA-NPs in both A549 and A549 DDP cells (Supplementary Fig. S7). It is important to note that although the CO1 and CO2 subunits were not altered in the gene expression levels after treatment with miR34a HA-NPs, we did observe changes in the complex IV activity. Similarly, the Complex I levels and activity were also elevated after treatment with miR34a HA-NPs in both A549 and A549 DDP cells (Supplementary Fig. S8). No significant major changes were observed after treatment with blank nanoparticles or naked miR34a. Hence, the changes in the mitochondrial epigenetic and transcription status were also translated into altered mitochondrial protein levels and release of CytC under the influence of miR34a HA-NPs.

### Lactate dehydrogenase (LDHA) levels and lactate production after treatment with miR34a HA-NPs

Since mitochondrial transcription was elevated by miR34a HA-NPs treatment, we wanted to measure if there was any change in glycolytic metabolism. Lactate levels are used as an indirect indicator of glycolysis^66^. We investigated if miR34a HA-NPs treatment (50 nM, 24 hrs) induced any changes in the lactate levels in A549 and A549 DDP cells. In comparison to untreated cells, the lactate levels were decreased about 1.5 fold after treatment with miR34a HA-NPs in A549 cells (p < 0.05). In contrast, there was a slight decrease in A549 DDP cell lines; however it was not significant (p = 0.07). Since LDHA converts pyruvate to lactate and is shown to have higher activity in cancer cells^41, 67^, we investigated underlying changes in LDHA mRNA levels. A549 and A549 DDP cells were treated with miR34a HA-NPs (50 nM, 24 hrs) and the mRNA levels of lactate dehydrogenase were measured using qRT-PCR. A significant (~2 fold; p < 0.05) decrease in LDHA mRNA levels was observed in A549 cell lines as compared to untreated cell lines. Although there was a trend towards decreased levels, but such downregulation of LDHA mRNA was not significant in A549 DDP cells (p = 0.0542).

### Measurement of glutathione based redox status post-treatment with miR34a HA-NPs

High metabolic rates and mitochondrial dysfunction leads to elevated intracellular ROS levels in cancer cells. Pharmacological stimulation of ROS production and/or depletion of protective reducing metabolites e.g. glutathione (GSH), or suppression of antioxidant enzymes, can lead to destabilization of mitochondria and induction of apoptosis^4, 5^. Hence, we measured the levels of reduced GSH and oxidized GSH (GSSG), as well as the redox capacity GSH/GSSG, after treatment with miR34a HA-NPs in A549 and A549 DDP cells. The GSH/GSSG levels were significantly decreased (~2-fold; p < 0.05) with miR34a HA-NPs in both cell types as compared to untreated cells (Fig. 7E).

### ATP levels in A549 and A549^DDP^ cells following treatment with miR34a HA-NPs

Lastly, mitochondria from A549 and A549^DDP^ cells were isolated following treatment with miR34a HA-NPs and the levels of ATP were measured as described previously^68^. Similarly, we also measured the cytosolic ATP levels. As depicted in Fig. 7D, the levels of ATP were elevated after miR34a HA-NPs (50 nM, 24 hrs) treatment in mitochondria from both A549 and A549 DDP cells as compared to untreated cells, although these changes were not statistically significant (p = 0.052). In contrast, the cytosolic levels of ATP were significantly elevated in both A549 and A549 DDP cells after treatment with miR34a HA-NPs (p < 0.05). This is consistent with the elevated activity of the complex I and IV.

## Discussion

**Figure 8:**
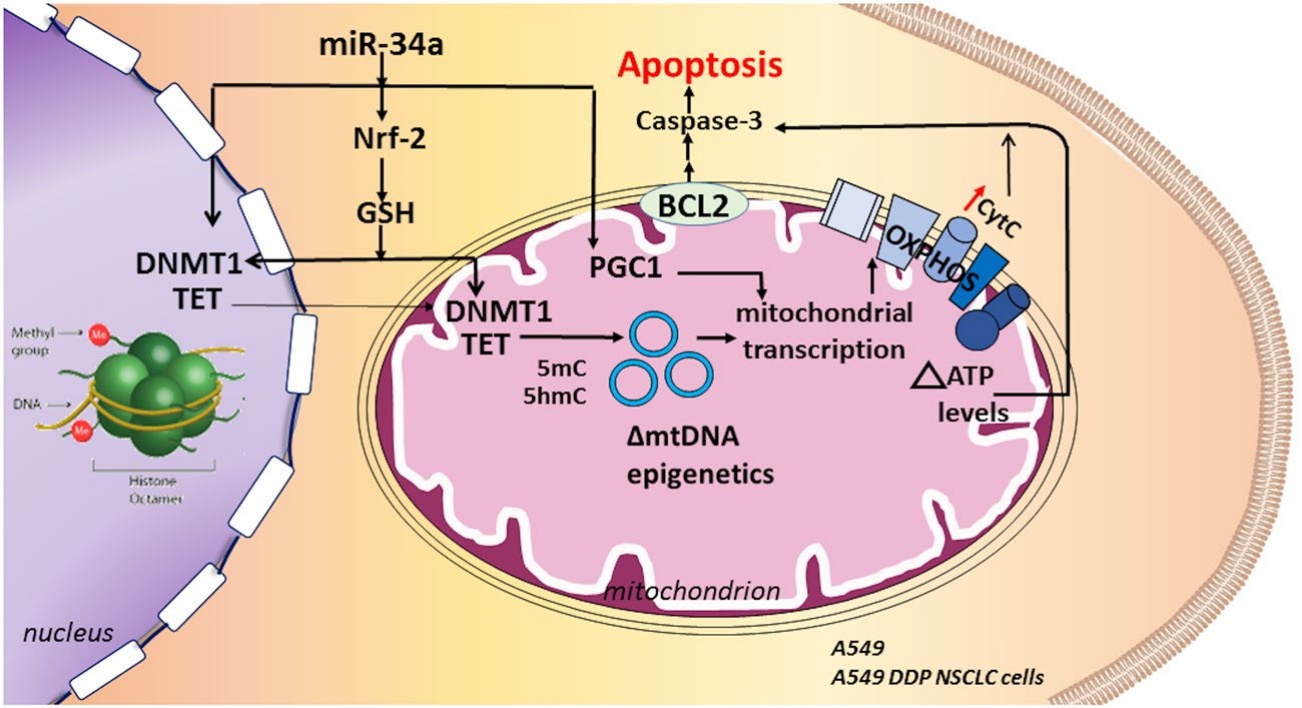
Summary Effect of miR34a. The miR34a delivery using HA-NPs can induce apoptosis in cisplatin sensitive (A549) and cisplatin resistant (A549 DDP) NSCLC cancer cell lines. miR34a can inhibit the Nrf-2 pathway and result in decreased levels of Glutathione (GSH). This altered redox status can further affect the levels of DNA methyl transferase (DNMT1) as well as Ten Eleven Translocase (TET) enzymes. Such altered levels of epigenetic enzymes can further lead to altered epigenetic status on mitochondrial DNA (mtDNA) and resulting in altered mitochondrial transcription and mitochondrial bioenergetics. Consequently, elevated mitochondrial ATP and cytochrome C oxidase can further contribute towards caspase activation and apoptotic induction. This process is also supported by the effects of PGC1-alpha on mitochondrial transcription and biogenesis. In parallel, miR34a can also inhibit the anti-apoptotic factor resulting in elevated levels of caspase-3 activation and apoptotic induction.

The use of small molecules and nucleic acid-based therapeutics for epigenetic reprogramming is limited. This is mainly due to its delivery using viral vectors; since there are several major limitations e.g. insertional mutagenesis, limited cargo capacity as well as immune toxicity concerns^39^. Here, we demonstrated that HA-PEI/HA-PEG-based nanoparticles could be successfully used for nucleic acid delivery for induction of apoptosis by altering the underlying metabolic and epigenetic status in cancer cells. Specifically, we report the efficient transfection of miR34a HA-NPs in wild-type and cisplatin-resistant A549 lung cancer cells that results in elevated oxidative stress leading to epigenetic changes, altered mitochondrial and cancer–cell bioenergetics ultimately contributing to apoptotic induction (Fig. 8).

Despite the heterogeneity of cells in tumors, almost all tumor cells demonstrate enhanced uptake and utilization of glucose, best known as the Warburg effect^3^. Such glycolytic ATP production indirectly results in mitochondrial dysfunction, enhanced mitochondrial stability and resistance to apoptosis; for example, through elevated Bcl-2 located on mitochondrial membrane; or elevated reactive oxygen species (which is supported by elevated GSH levels). P53 can induce apoptosis by decreasing the levels of Bcl-2 along with regulation of mitochondrial flux and mtDNA-encoded transcripts^29, 69^. However, in the current study we did not see any changes in the levels of p53 in the current study (Supplementary Fig. S9), which is in contrast to the previous study. Several factors including dose and duration of treatment, as well as time of measurement might contribute towards such differential effects. Hence, we believe that these apoptotic effects are primarily mediated by miR34a. This is not surprising since, the miR34a is known to be a direct inhibitor of antiapoptotic gene Bcl-2^70^; consistent with our current findings. Furthermore, miR34a HA-NPs decreased lactate production and the levels of lactate dehydrogenase enzyme; hence, it decreased the glycolytic flux. However, mitochondrial ATP production seemed to increase, but this change was not significant. Such elevated levels of ATP are implicated in induction of apoptosis^71^. Furthermore, elevated reactive oxygen species coupled with decreased antioxidant capacity result in impaired mitochondria functioning that triggers downstream apoptosis signaling as observed in the current study. Such changes couldalso be attributed to the decreased PGC1alpha that contributes to promote mitochondrial biogenesis.

The miR34a is also a direct inhibitor of the antioxidant response element NRF2^72, 73^, which when downregulated can result in decreased GSH status^56^. Here we show the role of miR34a on the induction of apoptosis by depleting such antioxidant GSH and altering the underlying levels of GSH/GSSG based redox status (Fig. 7E). This is consistent with the previous studies indicating the role of GSH/GSSG in apoptosis^74, 75^. In fact, pharmacological manipulation of the redox status and/or depletion of protective reducing metabolites e.g., GSH and/or suppression of antioxidant enzymes e.g. SOD, destabilizes the mitochondria, elevates oxidative stress and results in apoptotic induction^4, 5, 76, 77^. Generally used antitumor agents such as cisplatin, mitomycin C, vinblastine, doxorubicin, camptothecin also increase the cellular oxidative stress levels beyond the threshold for cancer cells subsequently resulting in apoptotic induction^78^.

It is noteworthy to mention that, the GSH levels also play an important role in the acquiring of cisplatin resistance in tumor cells^79^. Cisplatin can induce impaired mtDNA and mtRNA metabolism. Such altered mtDNA metabolism under the influence of cisplatin is associated with GSH levels^80^. Hence, we utilized the cisplatin sensitive and resistant cells for the current study and investigated if there were any underlying differences in the metabolism between the A549 and A549 DDP cells, along with any differential effects of miR34a on these two different cell lines. Consistent with the previous literature^72, 81^, the miR34a HA-NPs treatment resulted in decreased GSH levels in both the A549 and A549 DDP cell lines. This was also accompanied with decreased levels of Nrf2. Such inherent differences were also observed in the LDHA and lactate levels after treatment with miR34a HA-NPs between the cisplatin sensitive and cisplatin resistant A549 and A549 DDP cells. This is consistent with the previous findings which depict the role of LDHA levels for acquired platinum resistance in cancer^82, 83^.

Such induction of metabolic switch was further accompanied by underlying molecular switch in form of epigenetic reprogramming. In particular, miR34a HA-NPs treatment resulted in altered epigenetic status on nDNA with parallel changes in the mRNA levels of epigenetic enzymes including decreased levels of DNMT1 and elevated levels of TET1–3. The elevated levels of TET can be linked with higher oxidative stress induction, which has been linked with the oxidation of the 5-methylcytosine on DNA leading to 5-hydroxymethylation^49^. However, the role of such 5-hydroxymethylation marks in cancer is still not clearly established. Although, numerous studies have focused on targeting DNMT1 for treatment of cancer^45, 47, 84^ but this is the first study identifying the role of miR34a for regulation of DNMT1 as well as the TET1–3 in cancer. The mechanism of such miR34a regulation of epigenetic enzymes is not known, but similar inhibition of DNMT1 by other compounds has been shown to reverse cisplatin resistance in A549 cells^85^. However, we only observed methylation changes in 1 CpG site on L1-retrotransposon promoter with no resultant changes in mRNA levels of LINE-1. This is not surprising, since the use of L1-methylation, levels as a proxy for the global DNA methylation levels are still under debate and might not be representative of the genome-wide methylation status^51^. Cancer cells possess specific epigenetic marks permissive of tumor cell proliferation and escape apoptosis across the entire genome and such studies for characterizing the genome-wide epigenome marks will be highly important for the future studies to identify specific epigenetic loci that are altered under the influence of miR34a and that contribute to the metabolic switch and apoptotic induction.

In addition to nDNA epigenetic changes, miR34a HA-NPs treatment also altered epigenetic enzymes in mitochondria and epigenetic status on mtDNA; including decreased levels of D-loop CpG methylation. Differences in the epigenetic status of the D-loop between the wild-type *vs* cisplatin resistant A549 cells at the mtDNA levels were also observed (pos1 CpG site). This is not surprising, since such epigenetic differences have been previously associated with cisplatin treatment^85^. Hence, both metabolic (GSH and lactate) as well as molecular (epigenetic changes) were observed between cisplatin sensitive A549 vs cisplatin resistant A549 DDP cell lines. Previous studies indicate ATP6, ND6, ND1 and 12 S rRNA to possess altered methylation levels and altered mRNA transcriptional status in the absence of p53^23^. ATP6 levels encoded by mtDNA are decreased in prostate cancer, due to hypermethylation of the promoter region of ATP6 on mtDNA^86^. Transcripts of genes encoded by mtDNA have also demonstrated to be altered in tissue biopsies of breast cancer and ovarian cancer patients^8, 87, 88^. Although we did not investigate epigenetic status at each of these sites, differential mRNA expression levels of the subunits of Complex I, III, IV and V encoded on the mtDNA were observed after treatment with miR34a HA-NPs in both A549 and A549 DDP NSCLC cells. Furthermore, such treatment also resulted in altered translation of mtDNA-encoded protein Cytochrome C oxidase, which is located in the extra-mitochondrial location and when activated can result in induced caspase apoptotic pathway as observed under the influence of miR34a HA-NPs. However, it is important to note that CO1 and CO2 subunits of Cytochrome C oxidase levels were not all upregulated in A549 DDP cisplatin resistant cell lines. This is consistent with the previous study that identified altered cytochrome C oxidase subunits in cancer cells under the influence of doxorubicin resistance^89–91^. Similarly, the NRF1 gene was also not differentially expressed in the A549 DDP cisplatin resistant cell lines under the influence of miR34a; similar to previous studies indicating the effects of cisplatin resistance on the mitochondria related genes^92^. Hence, the current study puts forth an effect of miR34a on metabolic and molecular axis as outlined in Fig. 8.

Targeted tumor therapy has been a long quest and remains challenging, but the current study builds upon our previous understanding of delivering a diversified range of cargo using HA-PEI/HA-PEG self-assembling nanoparticles for delivery to the tumor tissue. It is important to mention that although we did not target the nanoparticles to mitochondria, microRNAs; especially miR34a can be translocated to the mitochondria or can also indirectly influence it’s effects on the levels of antioxidant metabolites as well as epigenetic enzymes. The main objective of the current study was to display the potentially novel application for the HA-PEI/HA-PEG nanoparticles to deliver microRNA and other such molecules to reprogram the molecular and metabolic frontiers of cancer cells including epigenetic status on nDNA as well as mtDNA. This can potentially promote a glycolytic to mitochondrial switch counteracting the Warburg effect and subsequently inducing apoptotic stimuli in lung cancer and such other tumors.

## Conclusions

We successfully used miR34a HA-NPs to mimic the effects of p53, and induce apoptosis in both wild-type and cisplatin resistant A549 cells. This apoptotic induction is mainly mediated by alterations in underlying molecular and metabolic coupling status in form of altered epigenetic status on mtDNA and nDNA. Such changes contributes towards metabolic switch from glycolytic energy metabolism to mitochondrial mechanisms, subsequently resulting in elevated oxidative stress, depleted GSH and induction of apoptotic signals and tumor cells death.

The major novel implications of the current study is to put forth some detail about the mechanistic underpinnings of contribution of mtDNA epigenetic changes in cancer development and progression and further studies are required for in depth characterization of mitochondrial epigenome in cancer development and progression as well as preclinical assessment of such epigenetic therapeutic modalities in relevant tumor models.

## Materials and Methods

### Materials

Sodium hyaluronate (HA) with an average molecular weight of 20 kDa was obtained from Lifecore Biomedical Co. (Chaska, MN). Poly(ethylene imine) (PEI MW 20,000 Da) was obtained from Polysciences Inc, (Warrington, PA). 1-Ethyl-3-[3-dimethylamino)propyl]carbodimide hydrochloride (EDC) and N-hydroxysuccinimide (NHS) were purchased from Thermo Fisher Scientific (Waltham, MA). Mono-functional poly(ethylene glycol)-amine (PEG_2K_-NH_2_, MW = 2000 Da) was purchased from Creative PEG Works, Inc. (Winston Salem, NC). Cisplatin was purchased from Fisher Scientific (St. Louis, MO). The Label IT intracellular localization kit was received from Mirus (Madison, WA). Primers specific for pro and anti-apoptotic genes were purchased from Eurofins MWG Operon (Huntsville, AL). Alexa flour 488 dye was purchased from Bioss Antibodies (Woburn, MA). Alexa Flour 488-anti-human CD44 antibody was purchased from BioLegend (San Diego, CA). miR34a was obtained from Origene.

### Cell lines

A549 human non-small-cell lung cancer (NSCLC) cell line was obtained from ATCC (Manassas, VA). The corresponding platinum-resistant cell line A549 DDP was obtained from Massachusetts General Hospital (Boston, MA). Both the cell lines were cultured in Dulbecco’s Modified Eagle’s medium (DMEM)/F12 (Carlsbad, CA) supplemented with 10% FBS, and 1% penicillin/streptomycin (100 U/mL) (Thermo Fisher Scientific, Waltham, MA) and grown at 37 °C, 5% CO_2_. As previously described^93^, cisplatin (final concentration of 2 μg/mL) was supplemented in the culture media for the A549 DDP cells to continue maintaining the drug resistance phenotype. Cisplatin was removed three days prior to any experiments with A549DDP cells.

### Formulation and characterization of miR34a duplexes-encapsulated HA-PEI/HA-PEG NPs

Combinatorial designed HA-PEI conjugates were prepared according to the protocol previously published^42^. For the synthesis of HA-PEG conjugates, 50 mg of maleimide-PEG-amine was added to EDC/NHS activated HA. For formulation preparation the HA-PEI, HA-PEG solutions (3 mg/ml) were prepared by dissolving the polymer in PBS pH 7.4. The HA-PEI/HA-PEG ratio with the duplexes was optimized in a ratio of 27:1. The average particle size, size distribution and zeta-potential of HA-PEI/miR34a NPs were measured using a dynamic light scattering (DLS) instrument (Malvern Zetasizer, Westborough, MA). Transmission electron microscopy (JEOL, JEM-1000, Tokyo, Japan) was performed to assess the morphology of the duplexes-loaded nanoparticles (NPs) as previously described^42^. The NPs were also run on 4% agarose gel to check percent encapsulation of the miRNA. miRNA encapsulation was confirmed by decomplexing HA-PEI/miR34a NPs with anionic polyacrylic acid (PAA) by mixing an equal volume of HA-PEI/miR34a and PAA using a vortex mixer. The strongly anionic PAA displaces the miRNA by electrostatically interacting with the cationic PEI. The decomplexed samples were then run on a 4% agarose gel to ensure the presence of intact miRNA bands.

### Intracellular uptake of miR34a HA-NPs in A549 and A549 DDP cells

To observe the cellular uptake, the miR34a was previously labeled with a green fluorescence dye (FITC) using a *Label* IT Intracellular Localization Kit (Mirus Bio, Madison, WA) and HA-PEI was also pre-labeled with a red fluorescence dye, Rhodamine (ThermoFisher Scientific, Waltham MA). A549 and A549 DDP cells were seeded at a density of 1.0 × 10^5^ cells/well in an 8-well chamber of a Lab-Tek II chamber slide and pre-incubated for 24 h at 37 °C and 5% CO_2_. Serum-free DMEM containing miR34a HA-NPs at an equivalent dose of 50 nM of DNA was added to each well, followed by incubation for 30 min and 6 hours at 37 °C. After incubation, the cells were washed thrice with PBS and fixed in formalin (4%) for 10 min at room temperature, followed by nuclear staining with 1 μg/mL of DAPI (supplier, country). Glass coverslips were then placed on glass slides. The cellular uptake of miR34a HA-NPs was imaged on a Zeiss confocal microscope (Carl Zeiss, Cambridge, UK). We also used MitoTracker™ (ThermoFischer Scientific) for tracking the mitochondrial localization in the cells.

### 5.5 Quantitative analysis of cellular uptake of HA-PEI/HA-PEGmiR34a nanoparticles

A549 and A549 DDP NSCLC cells at 80% confluency were plated in T25 flasks (500,000 cells per flask) and cultured at 37 °C and 5% CO_2_. The cells were then transfected with FITC labeled miR34a HA-PEI/HA-PEGNPs at 50 nM equivalent dose of miR34a for 30 min, 1, 2, 4 and 6 hrs. Post-transfection, these cells were fixed in 4% formalin, followed by incubation with bovine albumin serum (BSA) (3% w/v) at room temperature for 30 min. Finally, the cells were then washed three times with PBS for FACS analysis in FL1 channel using a BD FACScalibur instrument (San Jose, CA). The data were analyzed using BD Cell-Quest Pro^TM^ software.

### Isolation of nuclear and mitochondrial DNA from transfected A549 and A549 DDP cells

Following appropriate treatment, the A549 and A549 DDP cells were scraped into sucrose isolation buffer (250 mM Sucrose, 1 mM EGTA, 20 mM Tris pH 7.4) and disrupted by 10 strokes with a Dounce homogenizer. Samples were gently lysed by 20 up and-down passages through a 20 G syringe needle in 500 ml cold cell lysis buffer and then centrifuged at 700 g for 10 min to pellet the nuclei. The supernatant was transferred to a new tube and centrifuged again at 12,000 g for 15 min to pellet the mitochondria. For DNA isolation, both mitochondrial and nuclear pellets were re-suspendedsepar﻿ately in a lysis buffer with proteinase K and incubated at 37 **°**C overnight. DNA for the analysis of DNA methylation was harvested and isolated using the FitAmp^TM^ Blood & cultured Cell DNA Extraction Kit from EPIGENTEK^®^ as previously described^94^. Isolated DNA was quantified using a ND-1000 NanoDrop spectrophotometer (Thermo Scientific; Wilmington, DE).

### Detection of 5hmC and 5mC content in nDNA and mtDNA

The ELISA assays were conducted as described previously^24^. The 5hmC content of mtDNA and nDNA were measured by the Hydroxymethylated DNA Quantification Kit (Epigentek, Brooklyn, NY). Briefly, 100 ng of respective DNA was bound to a 96-well plate. The hydroxymethylated DNA was detected using its respective capture and detection antibodies and quantified colorimetrically by reading the absorbance at 450 nm in a microplate spectrophotometer (Bio-Rad, Model 550, Hercules, CA). For measurement of 5mC content, we used the corresponding Methylated DNA Quantification Kit from Epigentek. The results are expressed in units calculated according to the manufacturer’s manual.

### Bisulphite sequencing for measuring individual CpG methylation

The bisulfite conversion and pyrosequencing for LINE-1 on nDNA and D-loop region on mtDNA were conducted as described previously^56, 60^. Briefly, for bisulfite conversion, the extracted DNA (500 ng; concentration: 50 ng/ml) was treated with the EZ DNA Methylation-Gold Kit (Zymo Research, Orange, CA, USA) according to the manufacturer’s protocol. 30 uL of M-Elution Buffer (Zymo Research) was used for the final elution. Methylation of DNA was quantified with bisulfite treatment of DNA and simultaneous polymerase chain reaction (PCR) followed by pyrosequencing, using primers and conditions previously described^56, 60^. A 50-ml PCR was performed in 25 ml of GoTaq Green Master mix (Promega, Madison, WI, USA), with 1pmol biotinylated forward primer, 1pmol reverse primer, 50 ng bisulfite-treated genomic DNA, and water. Results were reported as the percent of the sum total of methylated and unmethylated cytosines that consisted of 5-methylated cytosines (%5mC). Additionally, non-CpG cytosine residues were used as internal controls to validate bisulfite conversion. The Pyrosequencing-based assay can evaluate individual measures of methylation at more than one CpG dinucleotide. All samples were subjected to a quality control check incorporated in the software, which evaluates the bisulfite conversion rate of any cytosine not followed by a guanine. LINE-1 Methylation levels were analyzed at three different CpG sites on LINE-1Hs family and three different CpG sites on D-loop of mtDNA. We also conducted technical replicates to ensure the replicability of the experiments.

### Quantitative Real-time PCR

After treatment, RNA was isolated using the RNAqueous −4PCR kit (Ambion) according to the manufacture’s protocol. Extracted RNA was treated with DNase and quantified using a ND-100 NanoDrop spectrophotometer. RNA was reverse transcribed to cDNA with random hexamer primer using the Transcriptor First Strand cDNA Synthesis kit (Roche) according to the manufacturer’s protocol. Quantitative Real-time PCR with previously designed primers using a Roche Light Cycler. Experimental conditions were same as previously described. Primer sets were checked for primer dimer formation and each primer was specific for the desired template. All assays were performed in triplicates and the triplicate CT values were averaged and normalized to the geometric mean of Beta-actin for nuclear genes and 18 s rRNA for mitochondrial genes, which were selected as endogenous controls based on previous preliminary experiments (data not shown). The normalized relative expression was calculated as Δ(ΔCT). CT values >36 were considered to be below the limit of detection.

### Isolation of intracellular reduced and oxidized glutathione (GSH and GSSG)

Following appropriate treatment and incubation, both A549 and A549 DDP cells were analyzed for their antioxidant status using HPLC as previously described^95^. Briefly, the media was aspirated and the cells were washed 2X with 1 mL of ice cold HBSS. HBSS was aspirated and 0.6 mL ice-cold dH_2_O was added to the cells. Cells were scraped from the flask/dish and suspended in the dH_2_O. The cell suspension was sonicated for 15 sec on ice and 100 µL of the sonicate was used to determine protein content. The remaining lysate was added to a microcentrifuge tube and an equal volume of 0.4 N perchloric acid was added, followed by incubation on ice for 5 min. Nitrogen gas was used to displace any oxygen in the sample tubes preventing any further oxidation of sample. These samples were then centrifuged at 13,000 RPM and the supernatant transferred to new microcentrifuge tubes. 100 uL of sample was added to a conical microautosampler vial and kept at 4 °C in the autosampler cooling tray. 10 uL of this sample was injected into the HPLC system.

The separation of reduced (GSH) and oxidized glutathione (GSSG) was accomplished using an Agilent Eclipse XDB-C8 analytical column (3 × 150 mm; 3.5 μm) and an Agilent Eclipse XDB-C8 (4.6 × 12.5 mm; 5 μm) guard column. Two mobile phases were used: Mobile Phase A was 0% acetonitrile, 25 mM sodium phosphate, 1.4 mM 1-octanesulfonic acid, adjusted to pH 2.65 with phosphoric acid. Mobile Phase B was 50% acetonitrile. The flow rate was initially set at 0.6 mL/min and a step gradient was utilized: 0–9 min 0% B, 9–19 min 50% B, 19–30 min 50% B. The column was then equilibrated with 5% B for 12 min prior to the next run. Temperature was maintained at 27 °C. The electrochemical detector wan an ESA CoulArray with BDD Analytical cell Model 5040 and the operating potential was set at 1500 mV. Sample concentrations were determined from the peak areas of metabolites using standard calibration curves and ESA-supplied HPLC software. Sample concentrations were normalized against protein content. In some cases samples were diluted in mobile phase as needed or up to 50 ul of sample were injected to assure that glutathione levels were within the range of the standard curve.

### ATP Analysis

Cell extracts were prepared post-transfection and an ATP colorimetric (Abcam, Cambridge, United States) assay was performed according to the manufacturer’s protocol.

### Lactate analysis

Cell extracts were prepared post-transfection and were used for measurement of lactate according to the manufacturers’ instructions (Lactate Colorimetric kit, Abcam). Lactate levels were normalized to cell numbers.

### ELISA assays (p53, Cytochrome C and NADH dehydrogenase)

Cell extracts were prepared post-transfection and were used for measurement of p53 levels, CytC levels or NADH dehydrogenase levels using ELISA based assay according to the manufacturers’ instructions (Abcam). These levels were normalized to the protein content.

### Caspase-3 activity assay

Caspase-3 activity was assessed using caspase-3 colorimetric assay kit (Abcam, USA) according to manufacturer’s protocol. Briefly, after the period of treatments, cells were trypsinized and centrifuged at 1100 g for 10 min. Cell pellets were suspended in 1 ml cold PBS and centrifuged at 4000 g for 5 min at 4 °C. In order to extract total protein, cell pellets were resuspended and lysed with 100 μl of chilled lysis buffer and incubated on ice for 10 min. Cell lysates were then centrifuged at 10000 g for 1 min at 4 °C. The concentration of proteins was measured by Bradford assay. Then 50 μl of 2x reaction buffer containing 10 mM DTT and 5 μl of 4 mM caspase-3 substrate (DEVD-*p* NA) were added to 200 μg protein from each sample and incubated at 37 °C for 4 h. The *p*-NA light emission was quantified using ELISA plate reader at 405 nm. Comparison of the absorbance of *p*-NA from an apoptotic sample with a control allowed determination of the fold increase in caspase-3 activity.

### Statistical analysis

Statistical analyses were performed using analysis of variance with the Bonferroni post hoc test or Student’s *t* test as appropriate. Differences were considered significant at *P* < 0.05. All statistical analyses were conducted using Prism 6.0 software (Graph-Pad Software, San Diego, CA).

## Electronic supplementary material

Supplementary information
