# Supplementary material for: MicroRNA-34a Encapsulated in Hyaluronic Acid Nanoparticles Induces Epigenetic Changes with Altered Mitochondrial Bioenergetics and Apoptosis in Non-Small-Cell Lung Cancer Cells
Source: Sci Rep. 2017 Jun 16;7:3636. doi: 10.1038/s41598-017-02816-8 (PMC5473901; doi:10.1038/s41598-017-02816-8)

MicroRNA-34a Encapsulated in Hyaluronic Acid Nanoparticles Induces Epigenetic Changes with Altered Mitochondrial Bioenergetics and Apoptosis in Non-Small-Cell Lung Cancer Cells

Malav Trivedi[1,2,*], Amit Singh[1], Meghna Talekar[1], Grishma Pawar[1], Parin Shah[1], and Mansoor Amiji[1,3]

[1] - Department of Pharmaceutical Sciences, School of Pharmacy, Northeastern University, Boston, Massachusetts, USA- 02115.

[2] - Department of Pharmaceutical Sciences, School of Pharmacy, Nova Southeastern University, Davie, Florida, USA – 33317.

[3] - Faculty of Pharmacy, King Abdulaziz University, Jeddah, Saudi Arabia.

* Corresponding Author
Malav S. Trivedi, PhD
Assistant Professor
Center for Collaborative Research
College of Pharmacy
Nova Southeastern University
Rm # 422, CCR building
3200 S. University Drive,
Fort Lauderdale, FL- 33317
Ph-954-262-1670
mtrivedi@nova.edu

Supplementary figure S1 HA-PEI/miR34a Uptake

Flow cytometry analysis indicated that the miR34a HA-NP'Ss were taken up by the A549 cells and cisplatin resistant A549 cells (A549 DDP) cells in a time dependent manner. The HA was conjugated to Rhodamine dye and the miR34a was conjugated with FITC.

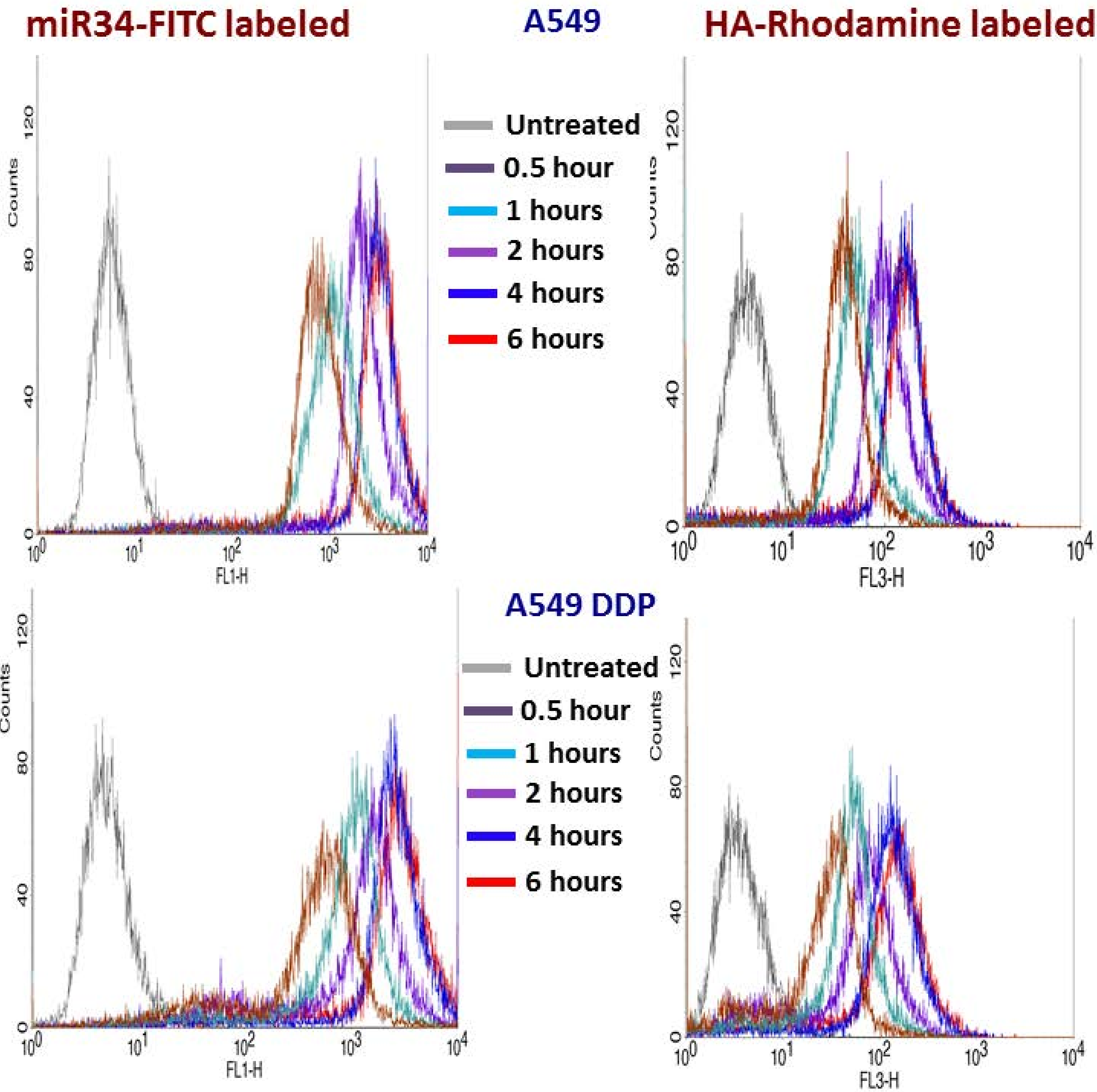

Supplementary Figure S2 HA-PEI/miR34a Uptake

Flow cytometry analysis indicated that the miR34a HA-NP'Ss were taken up by the A549 cells and cisplatin resistant A549 cells (A549 DDP) cells in a time dependent manner. The HA was conjugated to Rhodamine dye and the miR34a was conjugated with FITC.

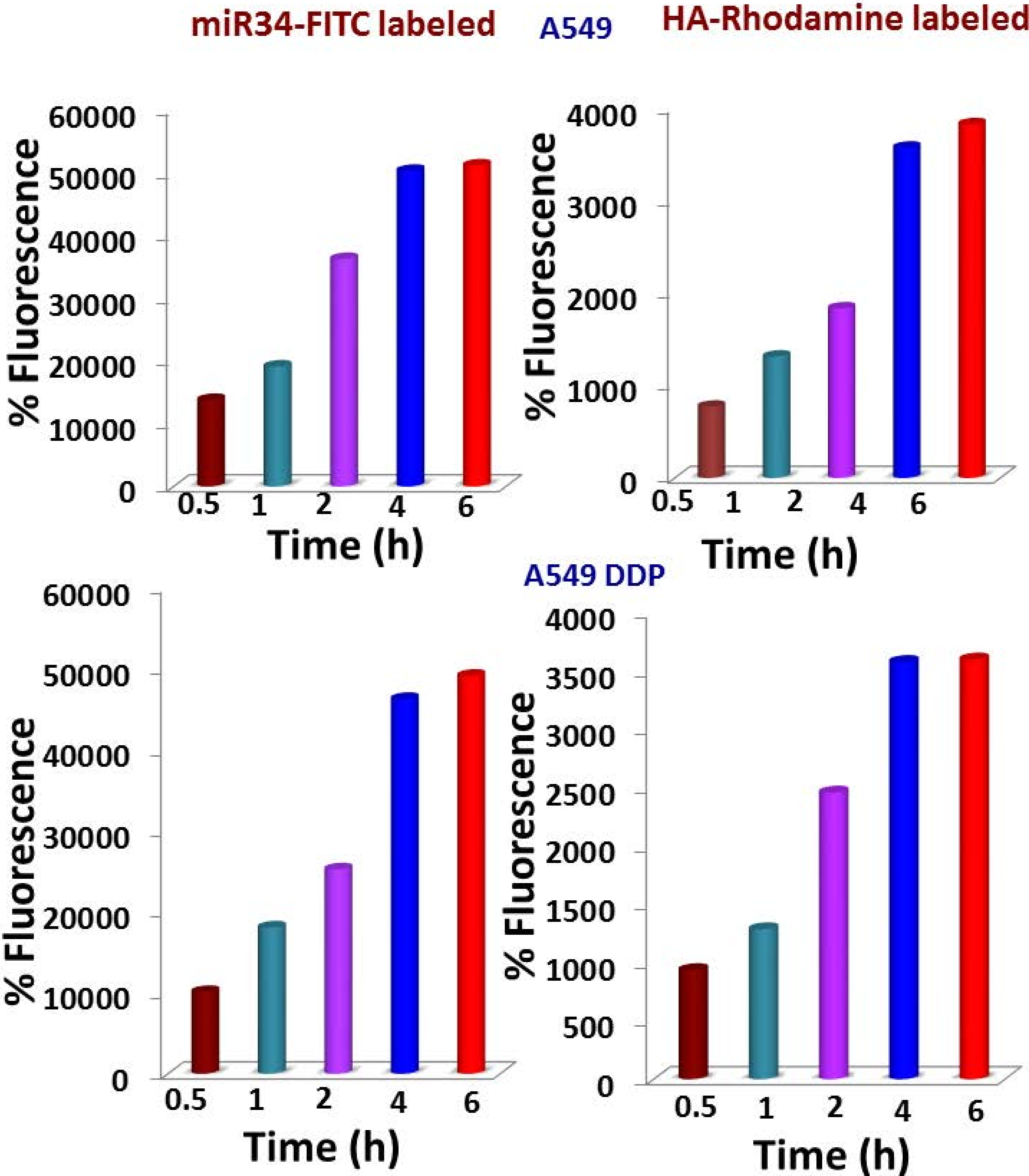

Supplementary figure S3 HA-PEI/miR34 Uptake: A549wt

Confocal images to support the flow cytometry results indicating that the FITC-miR34a encapsulated in HAPEI nanovectors

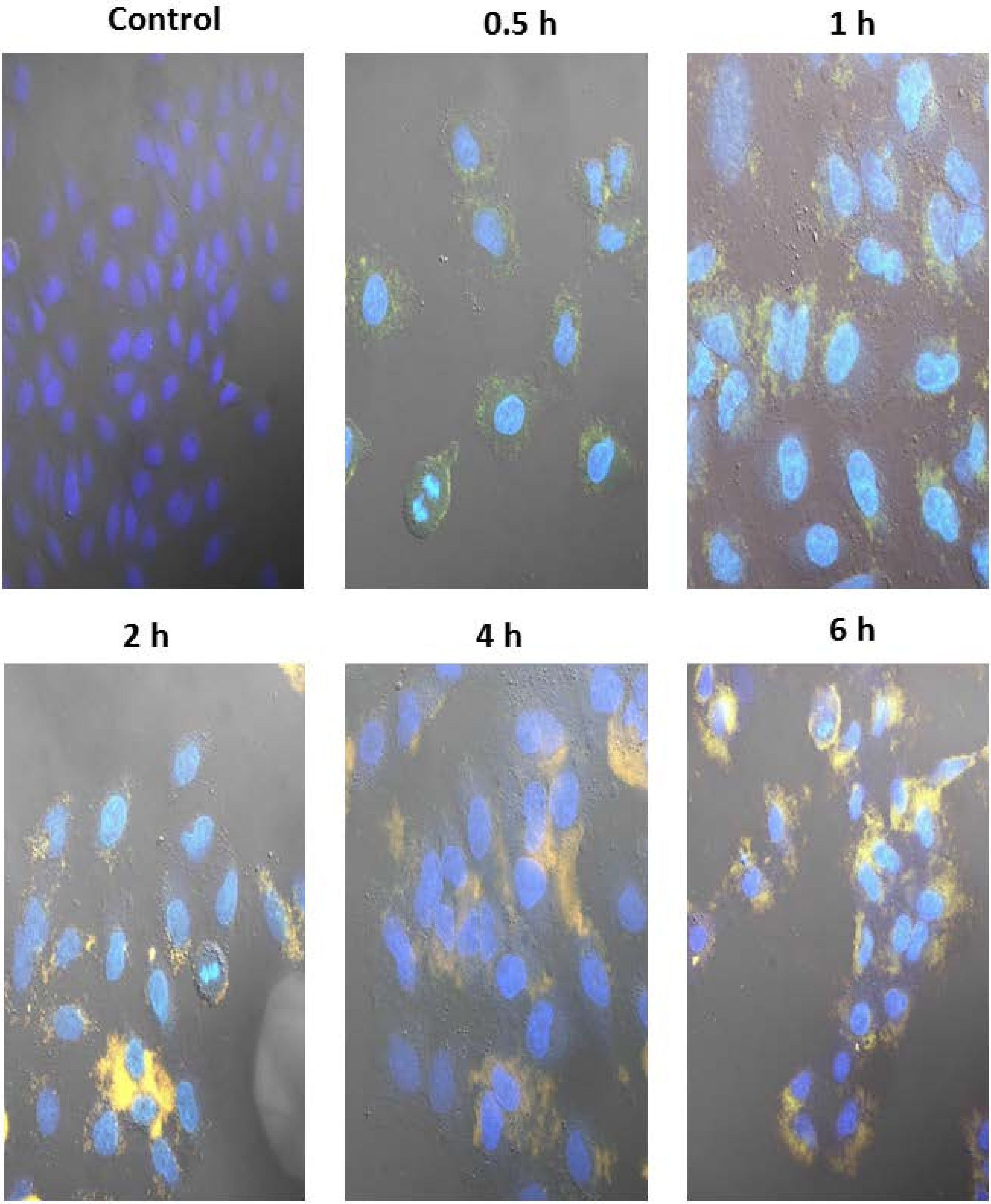

Supplementary figure S4 HA-PEI/miR34 Uptake: A549DDP

Confocal images to support the flow cytometry results indicating that the FITC-miR34a encapsulated in HAPEI nanovectors

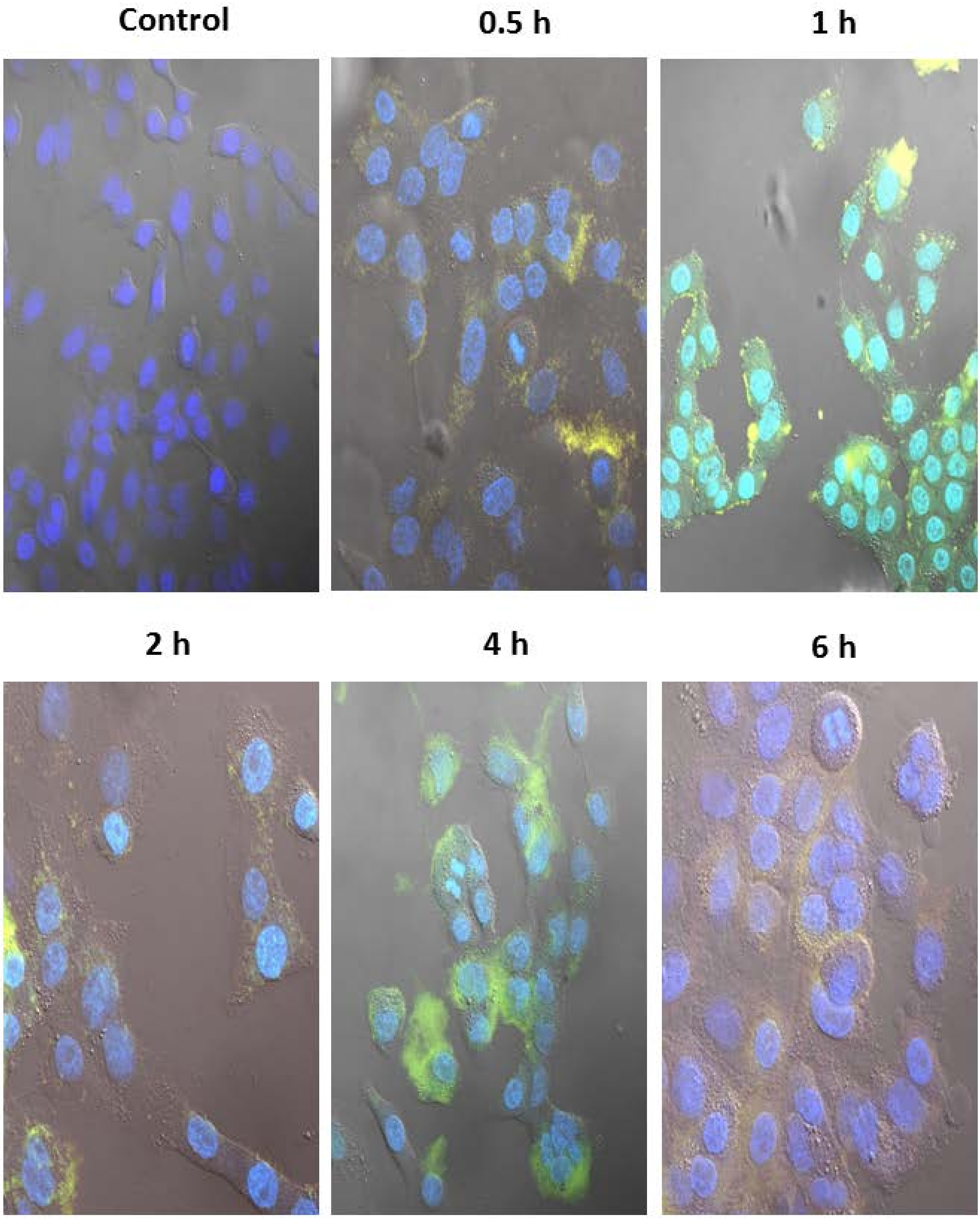

Supplementary figure S5 HA-PEI/miR34a/Mitotracker: A549wt

Confocal images to support the flow cytometry results indicating that the FITC-miR34a encapsulated in HAPEI nanovectors and mitochondria stained by MitoTrackr™.

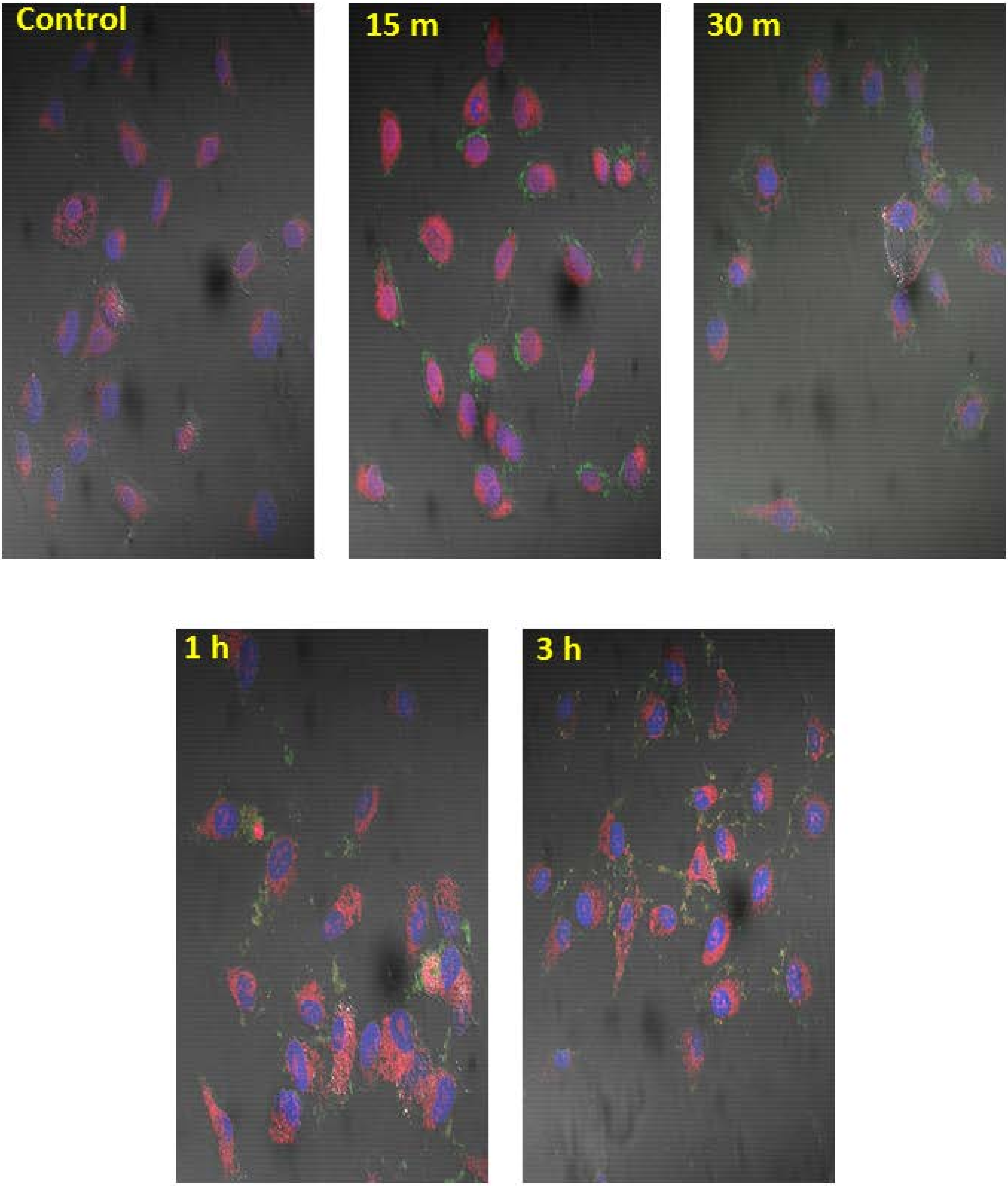

Supplementary figure S6 HA-PEI/miR34a/Mitotracker: A549^{DDP}

Confocal images to support the flow cytometry results indicating that the FITC-miR34a encapsulated in HAPEI nanovectors and mitochondria stained by MitoTrackr™.

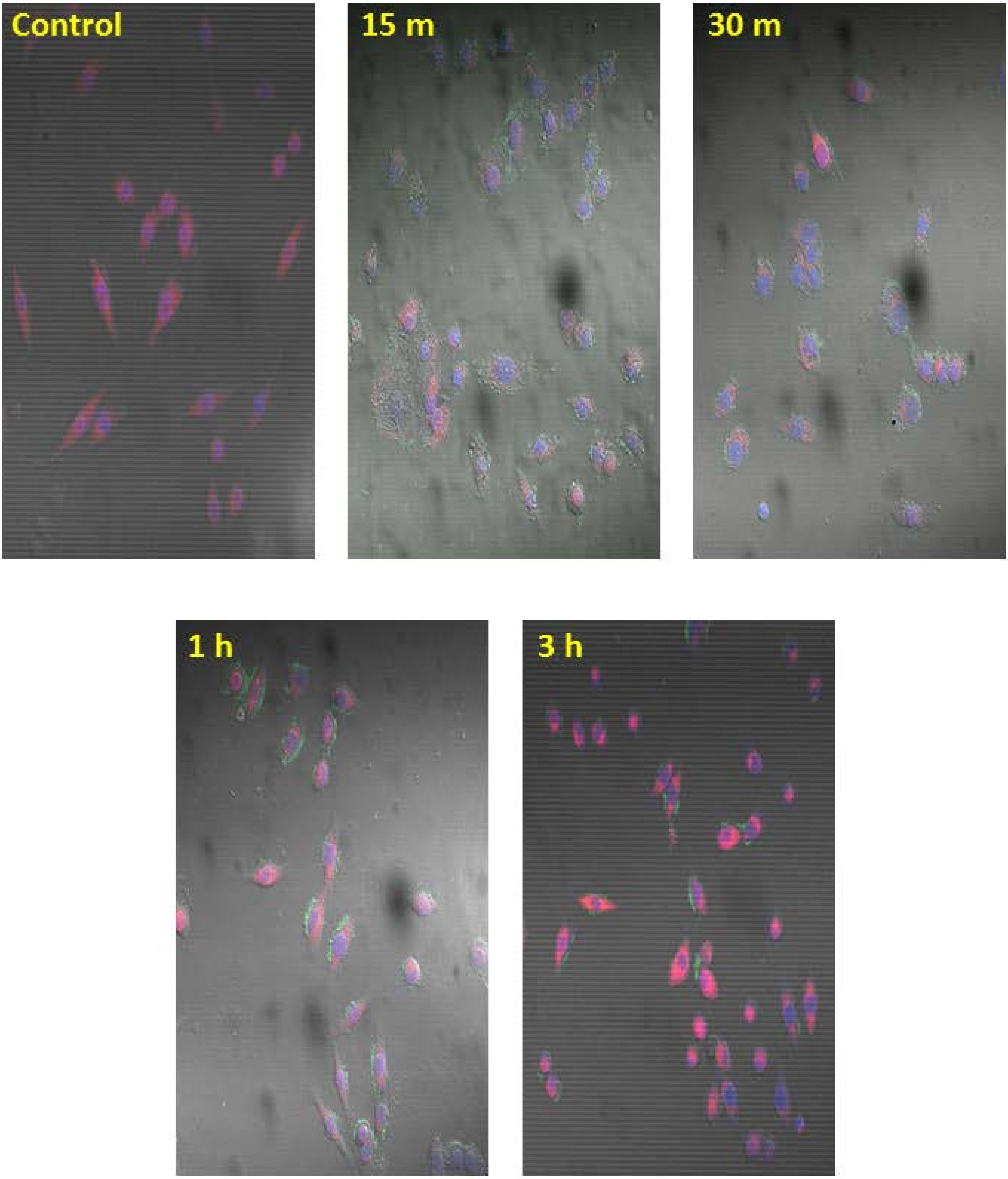

Supplementary Figure S7 Complex I (NADH dehydrogenase) levels and activity

Complex I levels (A and B) and enzyme activity (C and D) in A549 and A549 DDP cells , with and without treatment with miR-34a HA NPs. Data is represented as mean +/- SEM (N=5). * p<0.05 comparision vs untreated cells.

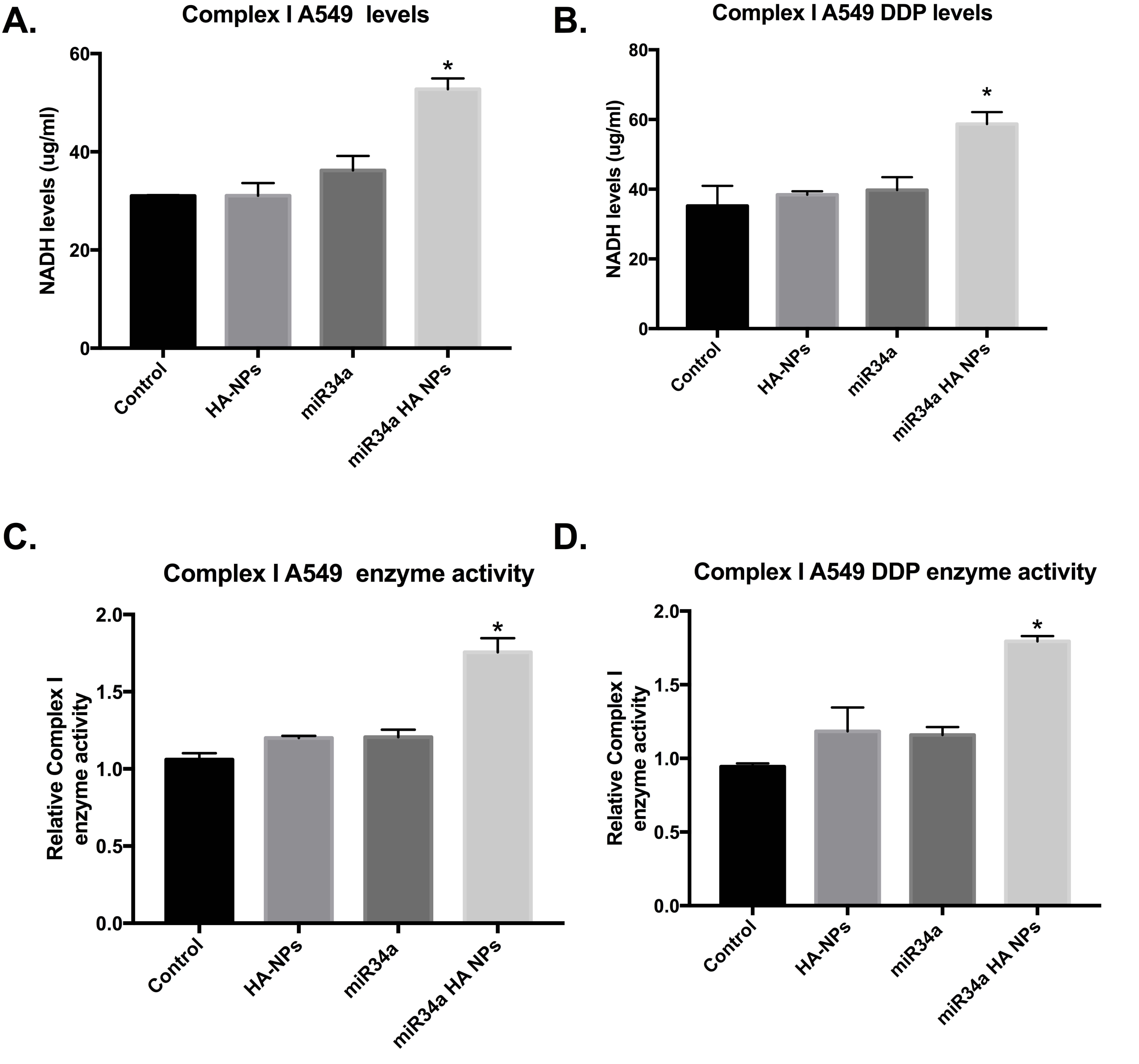

Supplementary Figure S8 Complex IV (cytochrome C) levels and activity

Complex IV levels (A and B) and enzyme activity (C and D) in A549 and A549 DDP cells , with and without treatment with miR-34a HA NPs. Data is represented as mean +/- SEM (N=5). * p<0.05 comparision vs untreated cells.

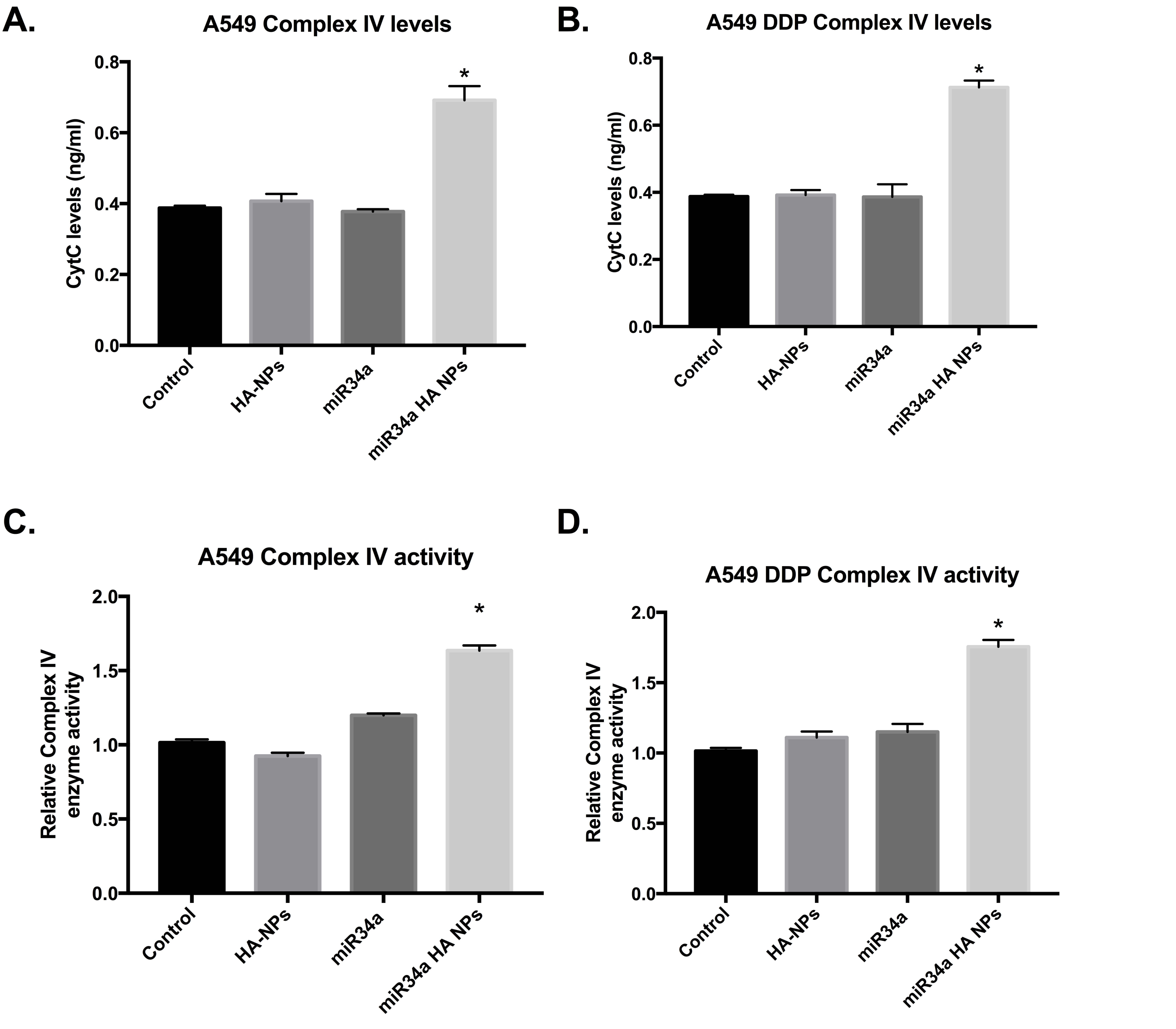

Supplementary Figure S9 p53 levels

(A) ELISA and (B) Western Blot based analysis of p53 levels in A549 and A549 DDP cells , with and without treatment with miR-34a HA NPs (N=5). data is represented as mean +/- SEM.

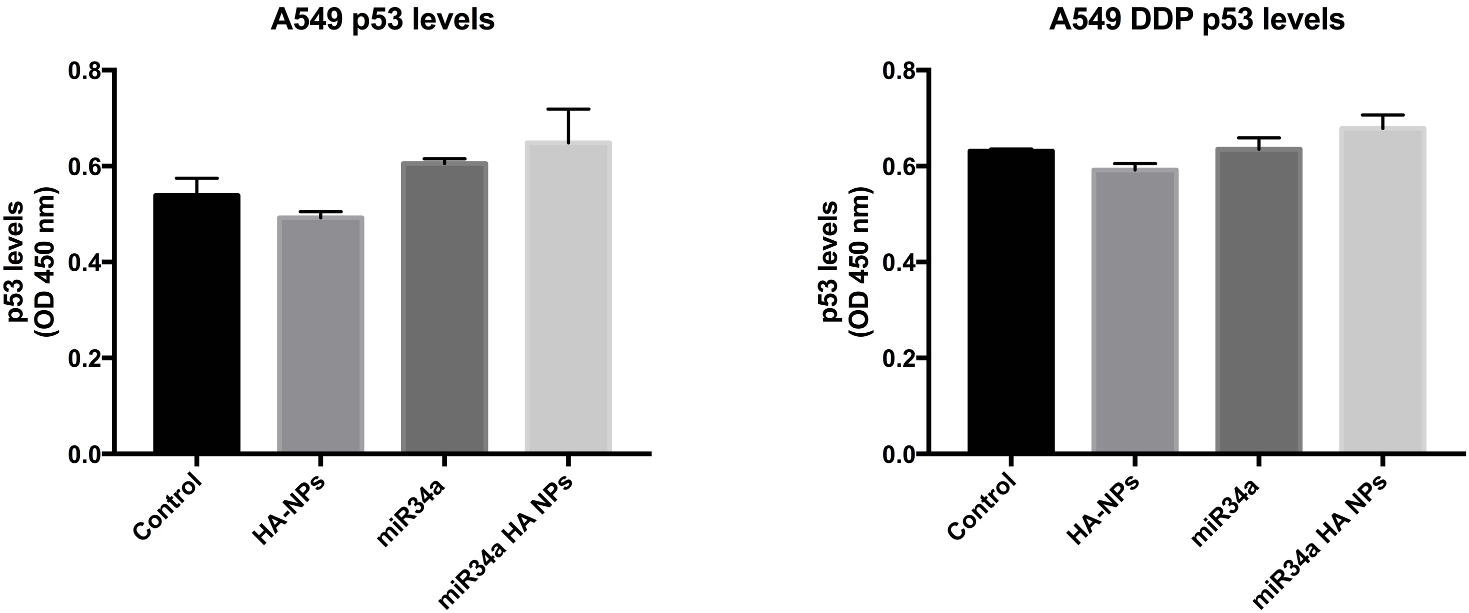

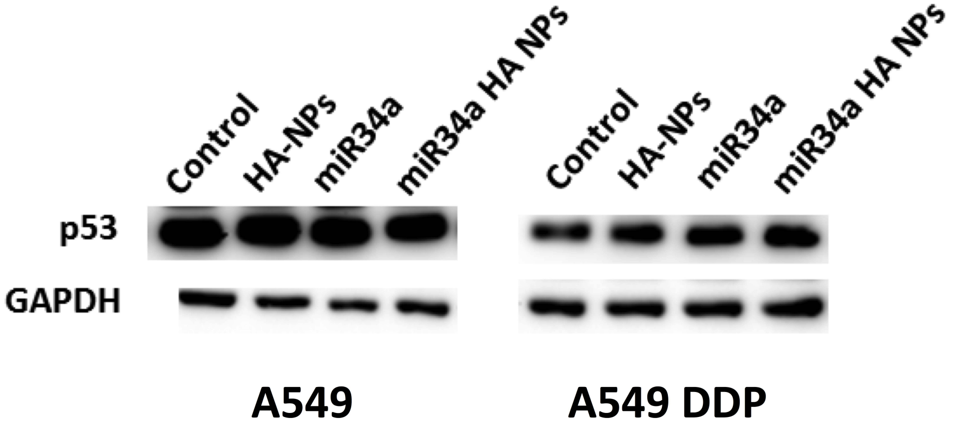

Supplementary Figure S10 Nanoparticle Characterization

(A). Size distribution of miR-34a HA-NPS nanoparticles in phosphate-buffered saline (pH 7.4) by dynamic light scattering. (B) The NP diameter from different batches of preparation ranged from 260 to 360nm, polydispersity index ranged from 0.2 to 0.3, and surface charge was in the range of −34 to −36 mV (C) Agarose electrophoretic analysis of miR-34a duplex encapsulation in HA-PEI NPs with polymer-to-miRNA weight ratios (27:1).

A.

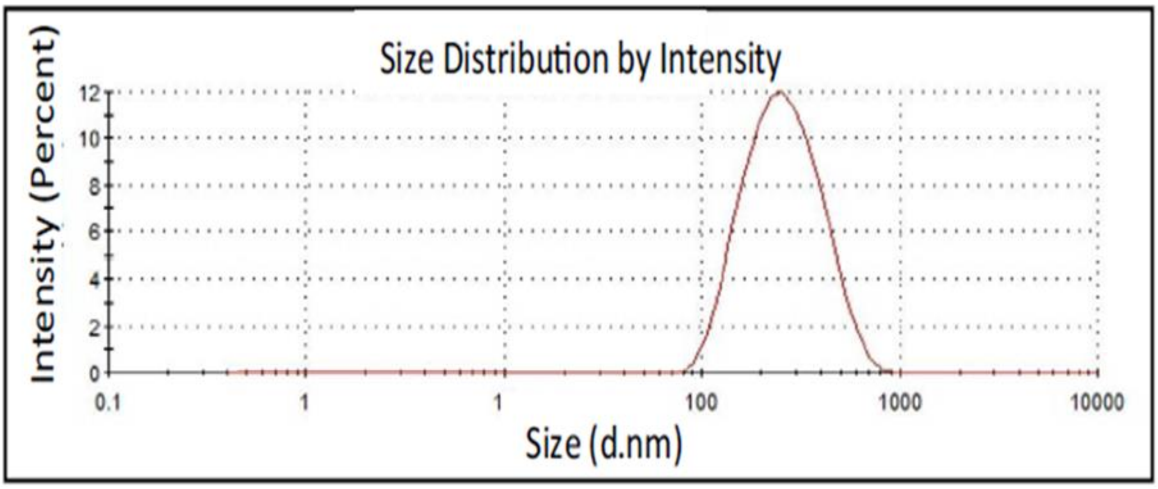

B.

Formulation	Size (d.nm)	PDI	Charge (mV)	Encapsulation efficiency (%)
HA-NPs	261±8	0.285	-34±3	95±3.6
HA-NPs miR-34a (Batch1)	363±17	0.276	-36±2	94±3.1
HA-NPs miR34a (Batch2)	346±15	0.249	-35±4	96±2.2

C.

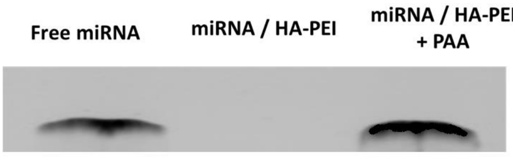

Supplement: Supplementary file 1 — Supplementary information [file 41598_2017_2816_MOESM1_ESM.pdf]
